# Patient‐Derived 3D Bioprinted Cardiac Organoid Constructs Reveal Key Pathological Features of Duchenne Muscular Dystrophy

**DOI:** 10.1002/adhm.202504004

**Published:** 2026-02-12

**Authors:** Vittoria Marini, Margalida Campaner Socias, Andreas Dimopoulos, Lorenza Rinvenuto, Enrico Pozzo, Diana Stalkopf, Angelo Serafini, Sara Morri, Ashley Wang, Rita La Rovere, Yoke Chin Chai, Sveva Bollini, Geert Bultynck, H. Llewelyn Roderick, Ioannis Papantoniou, Maurilio Sampaolesi

**Affiliations:** ^1^ Translational Cardiology Laboratory Stem Cell & Developmental Biology Unit Department of Development & Regeneration KU Leuven Leuven Belgium; ^2^ Skeletal Biology and Engineering Research Centre Department of Development & Regeneration KU Leuven Leuven Belgium; ^3^ Prometheus ‐ the Leuven R&D Translational Division of Skeletal Tissue Engineering KU Leuven Leuven Belgium; ^4^ Department of Radiotherapy and Radiosurgery IRCCS Humanitas Research Hospital Milan Italy; ^5^ Laboratory of Molecular & Cellular Signaling Department of Cellular & Molecular Medicine KU Leuven Leuven Belgium; ^6^ Interuniversity Microelectronics Center (imec) Leuven Belgium; ^7^ Department of Experimental Medicine (DIMES) University of Genova Genova Italy; ^8^ IRCCS Ospedale Policlinico San Martino Genova Italy; ^9^ Laboratory of Experimental Cardiology Department of Cardiovascular Sciences KU Leuven Leuven Belgium; ^10^ Histology and Medical Embryology Unit Department of Anatomy, Histology, Forensic Medicine and Orthopedics Sapienza University of Rome Rome Italy

**Keywords:** alginate, bioprinting, cardiac organoids, cardiomyopathies, Duchenne muscular dystrophy, gelatin

## Abstract

Duchenne muscular dystrophy (DMD) is a genetic disorder characterized by progressive muscle degeneration that significantly reduces the quality of life and lifespan of patients. Currently, cardiomyopathy represents the leading cause of death in later stages of the disease. While calcium dysregulation, fibrosis, and fat deposits are well‐documented hallmarks of DMD cardiomyopathies, the exact pathogenic mechanisms remain unclear due to the lack of reliable models. In this study, we developed 3D cardiac organoids (COs) from DMD patient‐derived induced pluripotent stem cells (DMD‐hiPSCs), their isogenic control (DMD‐Iso‐hiPSCs), and a healthy hiPSC line (HC‐hiPSCs). By day 15 of cardiac differentiation, DMD‐COs exhibited key disease features, including increased cell death, elevated ROS levels, and calcium signaling defects, compared with controls. Leveraging bioprinting technology, COs were embedded in a 7% alginate–5% gelatin hydrogel to generate bioprinted constructs (HC‐bCOs, DMD‐Iso‐bCOs, and DMD‐bCOs). These constructs self‐organized, displaying increased cell–cell communication, and reduced levels of the early cardiac transcription factor NKX2.5. By day 14 post‐bioprinting, DMD‐bCOs showed increased cell death and dysregulated expression of cardiac and fibrotic markers, mimicking DMD‐associated cardiomyopathy. This study demonstrates the potential of both COs and bCOs as a tool for studying DMD cardiomyopathy and advancing drug screening and therapies.

## Graphical abstract

Patient‐derived cardiac organoids reveal key features of Duchenne muscular dystrophy cardiomyopathy, including apoptosis, oxidative stress, calcium handling defects, and mechanical remodeling. By integrating organoids into alginate–gelatin bioprinted constructs, disease phenotypes are organized into scalable 3D cardiac tissues displaying extracellular matrix remodeling and fibrosis. This platform bridges organoid‐based disease discovery with tissue‐level modeling for translational applications.

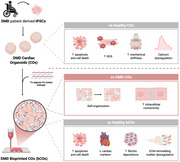

## 1 Introduction

Duchenne muscular dystrophy (DMD) is one of the most common muscular dystrophies, affecting approximately 1 in 3500–5000 live male births [1, 2, 3, 4, 5]. It is an X‐linked recessive genetic disorder caused by mutations in the DMD gene, which encodes the protein dystrophin. Dystrophin plays a critical role in maintaining the structural integrity of muscle cells, and its absence results in muscle degeneration, impaired regeneration, and progressive muscle weakness [1, 3, 4, 5]. While advancements in supportive care‐ such as respiratory assistance and non‐invasive ventilation‐ have significantly improved the life expectancy of DMD patients, this has also led to the emergence of cardiac complications, particularly dilated cardiomyopathy (DCM), which is now the leading cause of mortality in late‐stage DMD patients [1, 6].

DCM in DMD patients is characterized by progressive cardiomyocyte (CM) death driven by increased intracellular Ca^2^^+^ levels and elevated reactive oxygen species (ROS) leading to chronic inflammation, fibro‐fatty replacement of CMs, and ultimately, thinning of the left ventricular (LV) wall [1, 7, 8]. These changes result in reduced cardiac contractility and LV dilation, which eventually progress toward heart failure (HF) [1, 7, 9, 10]. Interestingly, Lu et al. identified a gene expression signature, termed “mesenchymal drift” (MD), which underlies several age‐related pathologies, including hypertrophic and DCM. Upregulation of this signature was shown to correlate with disease progression and increased mortality risk. However, the role of MD dysregulation in the context of DMD has not yet been investigated [11]. Despite its clinical importance, DMD‐associated DCM has not received significant research attention due to challenges including limited access to human cardiac tissue and the inability of conventional two‐dimensional (2D) CM cultures to replicate the complex three‐dimensional (3D) pathophysiology of human heart tissue [6, 7]. Moreover, although animal models like *mdx* mice and canine DMD models have provided insights into DMD progression, compensatory mechanisms and species‐specific muscle dynamics limit their ability to fully recapitulate late‐stage human disease, including cardiomyopathy. While canine DMD models develop cardiomyopathy similar to that seen in patients, their use is restricted by high costs and ethical considerations [7, 12].

The development of 3D cardiac organoids (COs) derived from patient‐specific human induced pluripotent stem cells (hiPSCs) offers a promising solution to bridge this research gap, enabling the generation of structurally complex, disease‐relevant CM models that retain the patient's genetic background. In addition, the use of CRISPR/Cas9 gene editing allows for the generation of precise isogenic controls [6, 13, 14, 15, 16]. However, standard organoid cultures partially lack the spatiotemporal biochemical cues and biomechanical signals essential for in vivo‐like tissue development. Bioengineered hydrogels can recreate key biophysical factors while supporting extracellular signaling that directs cell fate and tissue morphogenesis. In this context, 3D bioprinting can facilitate the creation of complex 3D cardiac structures by precisely depositing bioinks containing living cells and biomaterials in a layer‐by‐layer manner. This technique enables spatial organization and may facilitate better mimicry of native tissue architecture and signaling environments [16, 17, 18, 19]. In this context, bioprinting of spheroids and organoids has emerged as a standardized process enabling reproducible in vitro models [20, 21, 22, 23].

For cardiac tissue engineering, hydrogels derived from natural polymers such as gelatin, collagen, and alginate are commonly used to mimic the native extracellular matrix (ECM) and support CM adhesion, proliferation, and differentiation [16, 17, 24]. Gelatin, a biocompatible and biodegradable polypeptide derived from collagen hydrolysis, is widely utilized due to the presence of RGD motifs that promote cell attachment. Alginate, a bioinert copolymer extracted from brown algae composed of β‐*D*‐mannuronate and α‐*L*‐guluronate, offers excellent biocompatibility, tunable mechanical properties, and ion‐mediated cross‐linking, making it particularly suitable for extrusion and inkjet‐based 3D bioprinting and for applications requiring controlled stiffness and minimal degradation [25].

By embedding cardiac organoids within these biomaterials, organoid architecture and function can be enhanced, thereby obtaining in vitro constructs able to replicate the mechanical and structural properties of native cardiac tissue [17, 24].

Notably, bioprinted cardiac patches have shown promise in advancing personalized medicine by using hiPSC‐derived CMs, providing new avenues for both disease modeling and therapeutic applications targeting specific mutational landscapes [17, 18]. While 3D cardiac models have been used to study DMD cardiomyopathy, the application of organoid technology, particularly with proper isogenic controls, remains largely unexplored, especially in combination with bioprinting technologies [6, 24].

In this study, we explored the applicability of cardiac organoids (COs) and bioprinted cardiac organoids (bCOs) generated from DMD patient‐derived hiPSCs to investigate disease hallmarks and progression. Using this integrated approach, we recapitulated key features of DMD‐associated cardiomyopathy, including increased cardiomyocyte death and apoptosis, oxidative stress, calcium dysregulation, and fibrotic remodeling, thereby establishing these models as a relevant platform for studying DCM progression in DMD.

## 2 Results

### 2.1 Dystrophic Cardiac Organoids Exhibit Increased Cell Death, Oxidative Stress, and Mechanical Stiffness

**FIGURE 1:**
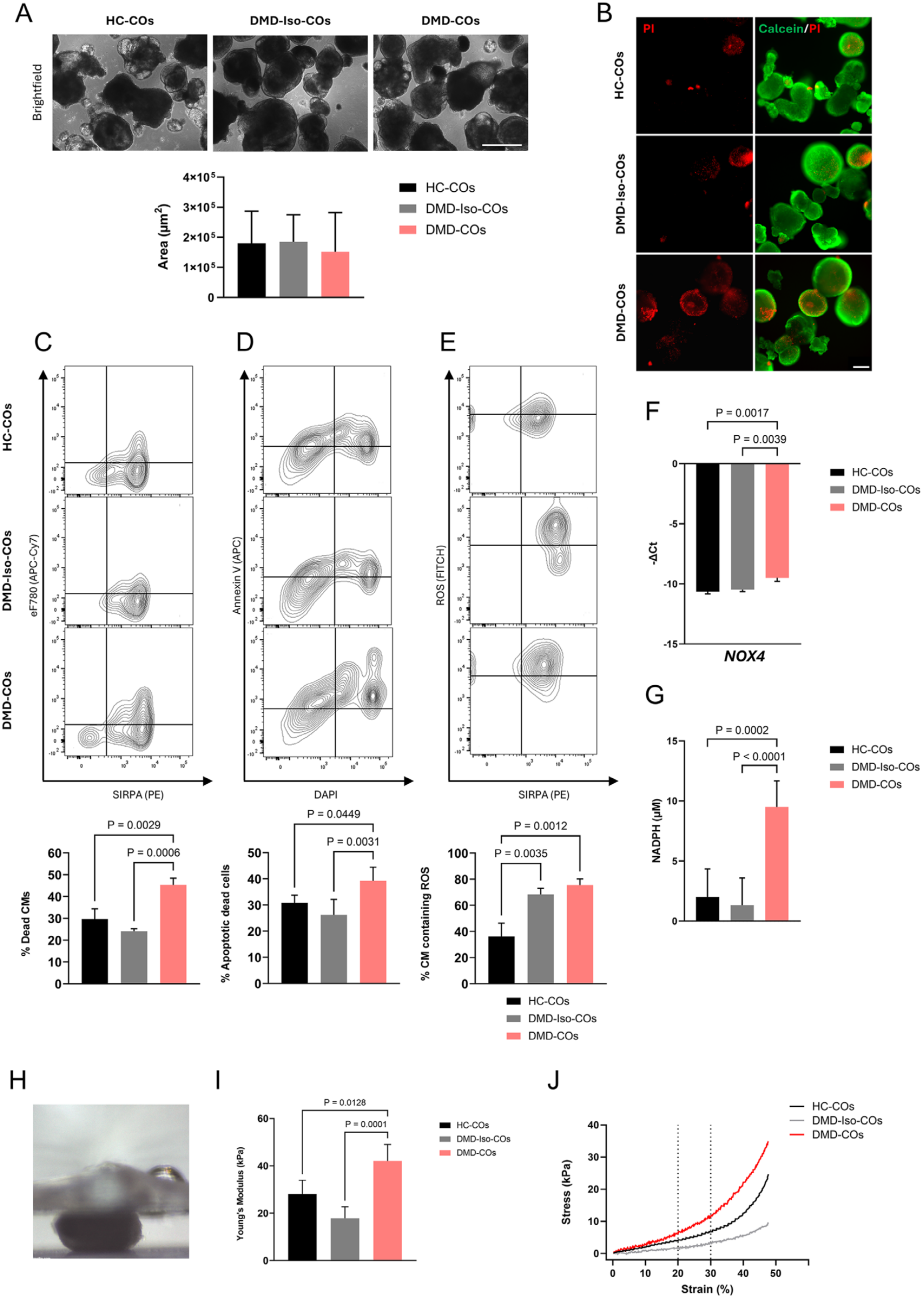
Increased premature cell death, apoptosis, and ROS production in DMD‐COs. (A) Representative brightfield images (top panel) and area quantification (bottom panel) of HC‐, DMD‐Iso‐, and DMD cardiac organoids (COs) at day 15 of cardiac differentiation. Scale bar = 500 µm. Data are representative of three independent experiments (*n* = 3) and expressed as mean ± SD. Statistical analysis was performed using one‐way ANOVA with Tukey's multiple comparisons test. (B) Representative live/dead staining images of HC‐, DMD‐Iso‐, and DMD‐COs at day 15 of cardiac differentiation. Calcein^+^ cells (green) = living cells; Propidium iodide^+^ cells (red) = dead cells). Scale bar = 250 µm. (C) Representative flow cytometric analyses (left panel) and quantification (right panel) at day 15 of cardiac differentiation showing the percentage of dead (eFluor780^+^) hiPSC‐CMs (SIRPA‐PE^+^) in HC‐, DMD‐Iso‐, and DMD‐COs. Data are representative of three independent experiments (*n* = 3, each pooled of ∼50 organoids) and expressed as mean ± SD. Statistical analysis was performed using one‐way ANOVA with Tukey's multiple comparisons test. (D) Representative flow cytometric analyses (left panel) and quantification (right panel) at day 15 of cardiac differentiation showing the percentage of dead (DAPI^+^) apoptotic (Annexin V‐APC^+^) cells in HC‐, DMD‐Iso‐, and DMD‐COs. Data are representative of five independent experiments (*n* = 5, each pooled of ∼50 organoids) and expressed as mean ± SD. Statistical analysis was performed using one‐way ANOVA with Tukey's multiple comparisons test. (E) Representative flow cytometric analyses (left panel) and quantification (right panel) at day 15 of cardiac differentiation showing the percentage of ROS‐containing (FITC^+^) hiPSC‐CMs (SIRPA‐PE^+^) in HC‐, DMD‐Iso‐, and DMD‐COs. Data are representative of three independent experiments (*n* = 3, each pooled of ∼50 organoids) and expressed as mean ± SD. Statistical analysis was performed using one‐way ANOVA with Tukey's multiple comparisons test. (F) Gene expression analysis of *NOX4* in HC‐, DMD‐Iso‐, and DMD‐COs at day 15 of cardiac differentiation. Each data point is represented as −Δ*Ct*, normalized to the housekeeping gene *GAPDH*. Data are representative of four independent experiments (*n* = 4, each pooled of ∼50 organoids) and expressed as mean ± SD. Statistical analysis was performed using one‐way ANOVA with Tukey's multiple comparisons test. (G) Quantification of NOX4‐dependent ROS production, measured via NADPH‐dependent ROS generation in HC‐, DMD‐Iso‐, and DMD‐COs at day 15 of differentiation. Data are representative of four or more independent experiments (*n* ≥ 4) and expressed as mean ± SD. Statistical analysis was performed using one‐way ANOVA with Tukey's multiple comparisons test. (H) Representative side‐view image of CO compression mechanical testing. (I) Average Young's modulus of HC‐, DMD‐Iso‐, and DMD‐COs at day 15 of cardiac differentiation. Data are representative of three independent experiments (*n* = 3) and expressed as mean ± SD. Statistical analysis was performed using one‐way ANOVA with Tukey's multiple comparisons test. (J) Representative stress/strain curves at day 15 of cardiac differentiation of HC‐, DMD‐Iso‐, and DMD‐COs.

Using our previously established protocol [6], we generated COs from healthy control (HC), isogenic DMD‐corrected (DMD‐Iso), and DMD‐, DMD #1‐, DMD #3‐, and DMD #5‐hiPSCs (Figure 1A; Figure S2A). After 15 days of cardiac differentiation, the cardiac identity of the COs was confirmed by the homogeneous distribution of sarcomeric cardiomyocyte markers, including α‐actinin and cardiac troponin T (cTnT) (Figure S1A). In addition to cardiomyocytes, other cell populations expressing α‐smooth muscle actin (α‐SMA), collagen type I alpha 1 (COL1A1), platelet endothelial cell adhesion molecule 1 (PECAM1), and perilipin (PLIN1) were detected across all conditions (Figures S1B,S2B). Notably, quantitative analysis revealed a significantly increased abundance of these non‐parenchymal cell types within DMD‐COs compared with HC‐ and DMD‐Iso‐COs (Figure S1B).

In order to assess the cell viability, we first performed a live/dead (Calcein‐AM/Propidium Iodide [PI]) staining at day 15 of cardiac differentiation (Figure 1B; Figure S2C), which revealed increased red PI fluorescence in dystrophic organoids, indicating loss of membrane integrity and subsequent inclusion of this dye by dead cells within the organoids.

To quantify the level of cell death, we conducted flow cytometric analysis on COs at day 15 of cardiac differentiation labelled with SIRPA‐PE antibody (surface CM marker) and eFluor780 to identify non‐viable cells based on membrane integrity. The results confirmed a significantly higher proportion of dead CMs in DMD‐COs compared to DMD‐Iso and HC. The two healthy lines, in turn, exhibited comparable levels of dead cells (Figure 1C). Further, flow cytometric analysis of COs revealed a marked increase in apoptotic dead cells (Annexin V^+^/DAPI^+^) in DMD‐COs relative to the other groups (Figure 1D). Additionally, we identified an increase in necrotic cells in DMD #1‐, DMD #3‐, and DMD #5‐COs compared to HC‐COs (Figure S2D).

To further investigate the molecular mechanisms driving CM pathological remodeling and death in COs, we analyzed bulk RNA‐seq data (GSE194297) of DMD‐ and DMD‐Iso‐COs at day 56 of cardiac differentiation and performed KEGG pathway enrichment analysis. DMD‐COs displayed upregulation of pathways related to cardiac remodeling, fibrosis, and adipose tissue deposition, which are known contributors to DMD‐associated cardiomyopathy (Figure S1C,D). The upregulation of a selection of key genes involved in these pathways further confirmed the activation of signatures related to cardiac remodeling, fibrosis, and adipogenesis in DMD‐COs compared to DMD‐Iso‐COs (Figure S1E).

Our group previously showed that DMD‐CMs have elevated intracellular ROS resulting from depolarized mitochondria and increased NADPH oxidase 4 (NOX4) [14]. To confirm these findings in our 3D environment, we first assessed ROS levels in DMD‐COs and their respective controls in organoid homogenates at day 15 of cardiac differentiation. (Figure 1E; Figure S3A). Both DMD‐ and DMD‐Iso‐COs exhibited significantly elevated ROS levels compared to HC, with no significant difference observed between DMD and isogenic COs. Subsequently, we investigated the expression of *NOX4* and *NOX2* and their regulatory subunits (*p22phox*, *p47phox*, *RAC1*, *RAC2*, and *RAC3*) in our 3D cardiac organoid models. Prior to analysis, we confirmed the stable expression of five commonly used housekeeping genes across our samples (Figure S3B), supporting the use of *GAPDH* for normalization (Figure S3B). At day 15, real‐time quantitative polymerase chain reaction (RT‐qPCR) analysis revealed a significant increase in *NOX4* mRNA in dystrophic COs when compared to COs generated from each of the control cell lines (Figure 1F; Figure S3C). Conversely, we found a downregulation of *NOX2* expression in DMD‐, DMD #1‐, and DMD #3‐COs in comparison to HC‐COs. In contrast, *p47phox* levels were increased in DMD‐COs, while *RAC1* expression showed a significant dysregulation in DMD‐ and DMD #3‐COs (Figure S3D).

Next, to assess whether elevated *NOX4* abundance corresponded to increased NOX4 activity, we measured NADPH levels using a colorimetric assay that detects the NADPH concentration in the organoid supernatant (Figure 1G). Particularly, NADPH levels in DMD‐COs reached up to 10 µm, significantly higher than in control organoids, which remained below 5 µm.

It has been documented that, as DMD progresses, the cardiac muscle becomes replaced by fibrotic tissue increasing the overall stiffness. This can, in turn, affect the mechanical properties of the cardiac organoids. Hence, to further corroborate the COs capacity to recapitulate this key feature of DMD cardiomyopathy, we conducted mechanical characterization through the compression of COs from all the tested conditions (Figure 1H). DMD‐COs demonstrated a significantly higher Young's modulus as well as higher compression stress compared to control organoids. (Figure 1I,J; Figure S2E).

Together, these findings highlight the ability of our dystrophic COs to recapitulate key molecular features related to DMD cardiomyopathy, such as enhanced cell death and NOX4‐mediated ROS production, together with an upregulation of remodeling‐associated genes, and an increase in organoid stiffness.

### 2.2 Calcium Handling Dysregulation in DMD‐COs

ROS perturbation and calcium dysregulation are closely interconnected in cardiomyocyte pathophysiology [7, 8]. To investigate potential impairments in calcium signaling in our dystrophic 3D cardiac models, we conducted an analysis of calcium dynamics using Cal520 dye and the FDSS/µCELL 96‐well imaging system from Hamamatsu Photonics, which monitors whole organoid fluorescence changes over time. The analysis was performed to capture spontaneous Ca^2+^ transients (unstimulated COs) and those provoked by electrical field stimulation at 0.5 and 1 Hz.

**FIGURE 2:**
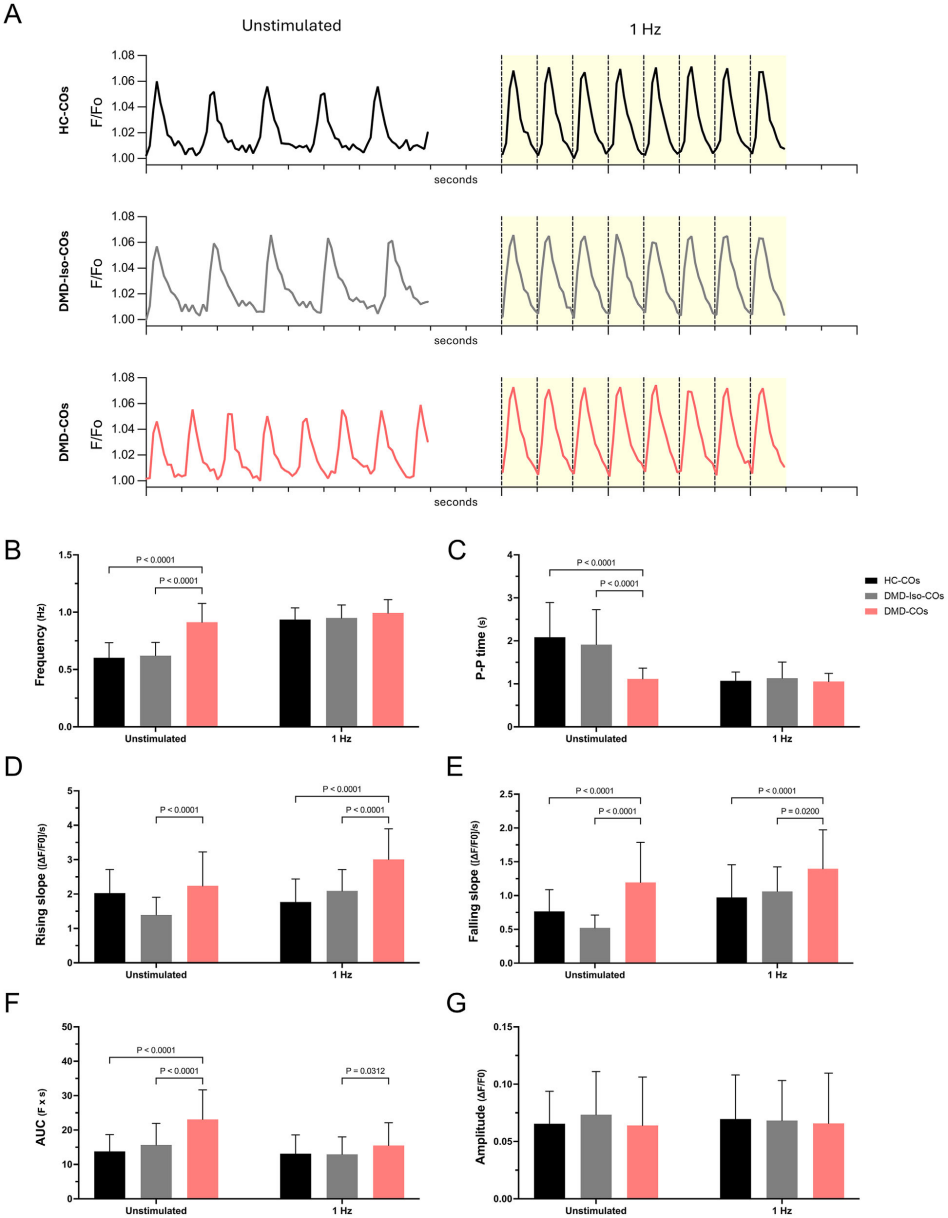
Dysregulated calcium handling in DMD‐COs. (A) Representative traces of Ca^2^^+^ transient recordings from Cal520‐loaded HC‐, DMD‐Iso‐, and DMD‐COs at day 15 of cardiac differentiation, plotted as *F*/*F*_0_(*F*_basal_) over time. Measurements were performed under unstimulated conditions and during electrical pacing at 1 Hz. (B–G) Quantification of calcium transient parameters, including: B) frequency, C) peak‐to‐peak (P–P) time, D) rising slope, (E) falling slope, (F) area under the curve (AUC), and (G) amplitude of HC‐, DMD‐Iso‐, and DMD‐COs on day 15 of differentiation under unstimulated and paced (1 Hz) conditions. Data are representative of three or more independent experiments (*n* ≥ 3) and expressed as mean ± SD. Statistical analysis was performed using one‐way ANOVA with Tukey's multiple comparisons test.

Figure 2A and Figure S4A illustrate traces of cytosolic Ca^2+^ dynamics across these conditions as *F/F_0_* (*F*_basal_), which corresponds to the lowest F values in the recording. All COs display spontaneous Ca^2+^ signaling, underpinning their physiological activity. In the unstimulated condition, HC‐ and DMD‐Iso‐COs exhibited rhythmic Ca^2+^ transients at ∼0.6 Hz, while the DMD‐COs displayed a higher frequency (∼0.9 Hz, Figure 2B). Consequently, electrical stimulation at 0.5 Hz was insufficient to standardize the beating frequency of the organoids across the different conditions and led to artificially induced events resembling early after‐depolarization events (Figure S4B). Conversely, when paced at physiological levels (1 Hz), COs derived from all cell lines successfully followed the pacing stimulus, indicating preserved responsiveness to external pacing (Figure 2B).

We then quantified additional key Ca^2+^ transient parameters, including peak‐to‐peak time (P–P time), both rising and falling slopes, area under the curve (AUC), and amplitude (Figure 2C–G; Figure S4C–H). DMD‐COs exhibited significantly higher rates of rise and fall of the Ca^2+^ transient compared to their healthy and isogenic counterparts across all conditions. (Figure 2D,E; FigureS4E,F). Similarly, AUC was significantly increased in DMD‐COs compared to the controls, at both baseline and 0.5 Hz, and only relative to the isogenic control at 1 Hz stimulation, although showing the same trend with HC‐COs (Figure 2F; Figure S4G). However, we found no significant differences in Ca^2+^ transient amplitudes in COs, either when unstimulated or paced (Figure 2G; Figure S4H).

To gain insight into the molecular basis of these Ca^2+^ handling differences, we analyzed bulk RNA‐seq data (GSE194297). DEG analysis confirmed significant dysregulation of markers related to action potential, Ca^2+^‐handling, and the reactivation of fetal genes in DMD‐COs compared to DMD‐Iso‐COs (Figure S4I–K). The observed landscape of upregulated (*RYR2*, *SERCA, NCX*, in green) and downregulated (*NCLX*, in red) Ca^2+^ transporters and channels, suggesting impaired Ca^2+^ cycling, indicates deregulated calcium dynamics, a key aspect of DMD disease. Together, these findings corroborate Ca^2+^ handling dysregulation in DMD‐COs, further establishing its connection to oxidative stress and cardiomyopathy progression.

### 2.3 Alginate–Gelatin Bioink Synthesis and Characterization

**FIGURE 3:**
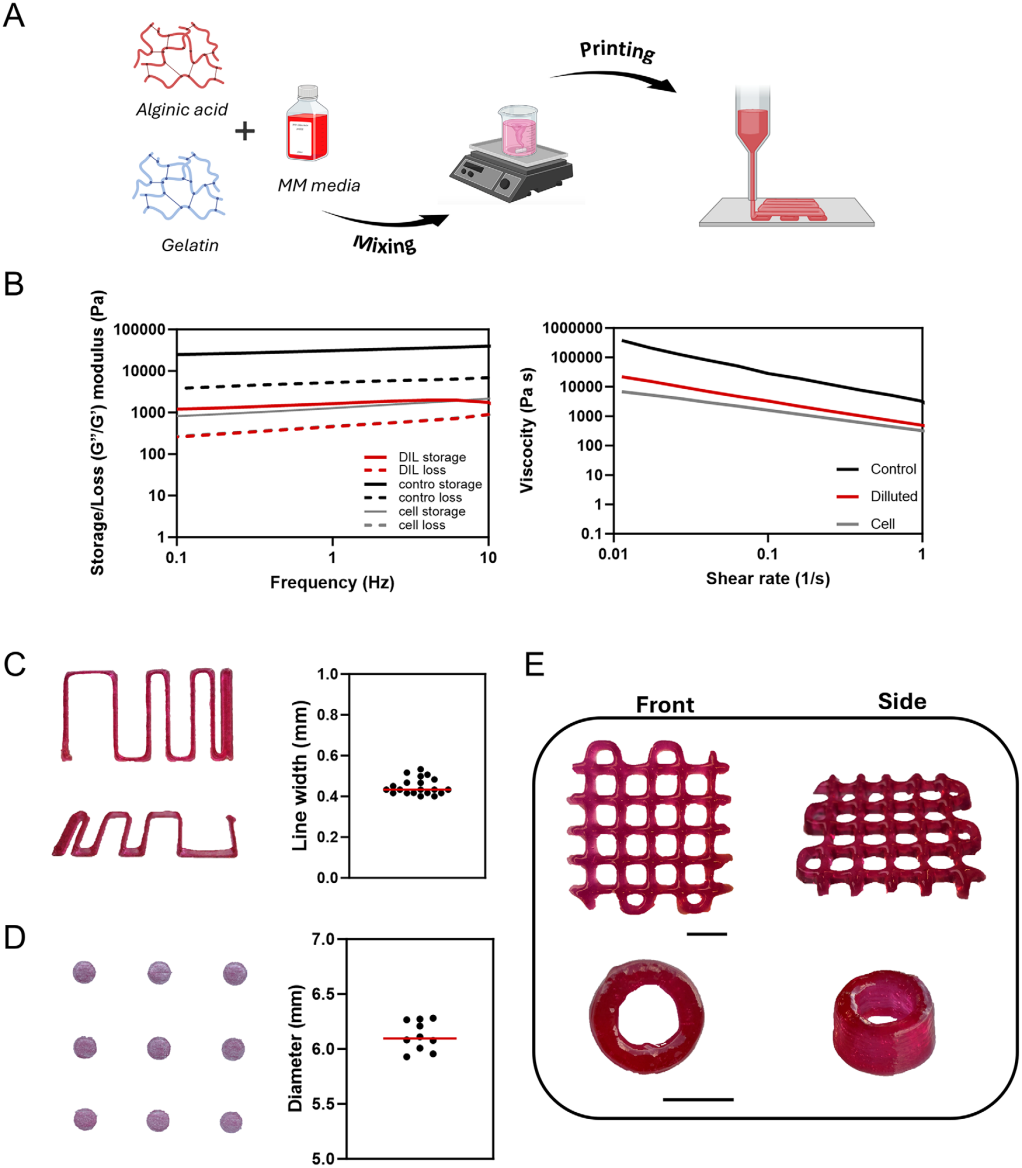
Synthesis and characterization of alginate/gelatin ink. (A) Illustration of gelatin/alginate ink synthesis. (B) Rheological characterization analysis of Storage (G′)/ Loss (G″) modulus and Viscosity for all the tested conditions. (C) Resolution line width analysis (*n* = 20). (D) Average diameter measurement (*n* = 9). (E) 3D bioprinted complex constructs demonstrate structural integrity post‐printing. Scale bar = 5 mm.

To more closely mimic the native ECM and support CM cell adhesion, a copolymer composed of 7% alginic acid and 5% of gelatin was developed by mixing the two polymers with MM media under stirring conditions (Figure 3A).

Rheological measurements were conducted for three formulations: alginate/gelatin ink alone (Control), ink diluted 1:3 with medium (Diluted) and ink mixed 1:3 with medium containingcardiac organoids to generate the cell‐laden bioink (Cell). Changes in the storage (G′) and loss (G″) modules were observed between the control and cell‐laden conditions. In parallel, higher relative viscosity was observed for the control group. Remarkably, there were no significant changes in the storage/loss modulus as well as the viscosity between the cell‐laden and the diluted condition (Figure 3B). We then conducted resolution analysis post‐printing by line width characterization and average diameter measurements of the diluted condition. The line width results demonstrated 0.44 mm with an average error of 0.04 mm (Figure 3C). The post‐printing analysis of cylindrical constructs with a diameter of 6 mm showed high accuracy as the analyzed average diameter was found to be 6.1 ± 0.13 mm (Figure 3D). The diluted ink was then loaded into the bioprinter, and complex 3D structures that required high accuracy and precision were printed. Post‐printing, the constructs showed structural fidelity after being printed without support or post‐processing, in a single extrusion step, maintaining their structural form (Figure 3E).

### 2.4 Alginate–Gelatin Hydrogel Promotes Organoid Fusion While Retaining the Beating Phenotype

We sought to incorporate COs into hydrogel to achieve more structurally complex models. Thus, HC‐COs were encapsulated at day 9 of differentiation in a 3% alginate‐ 2% gelatin hydrogel to create the bioink. To confirm the biocompatibility of the matrix composition in supporting CO viability and promoting organoid fusion, constructs were maintained in culture and monitored over 20 days post‐embedding.

**FIGURE 4:**
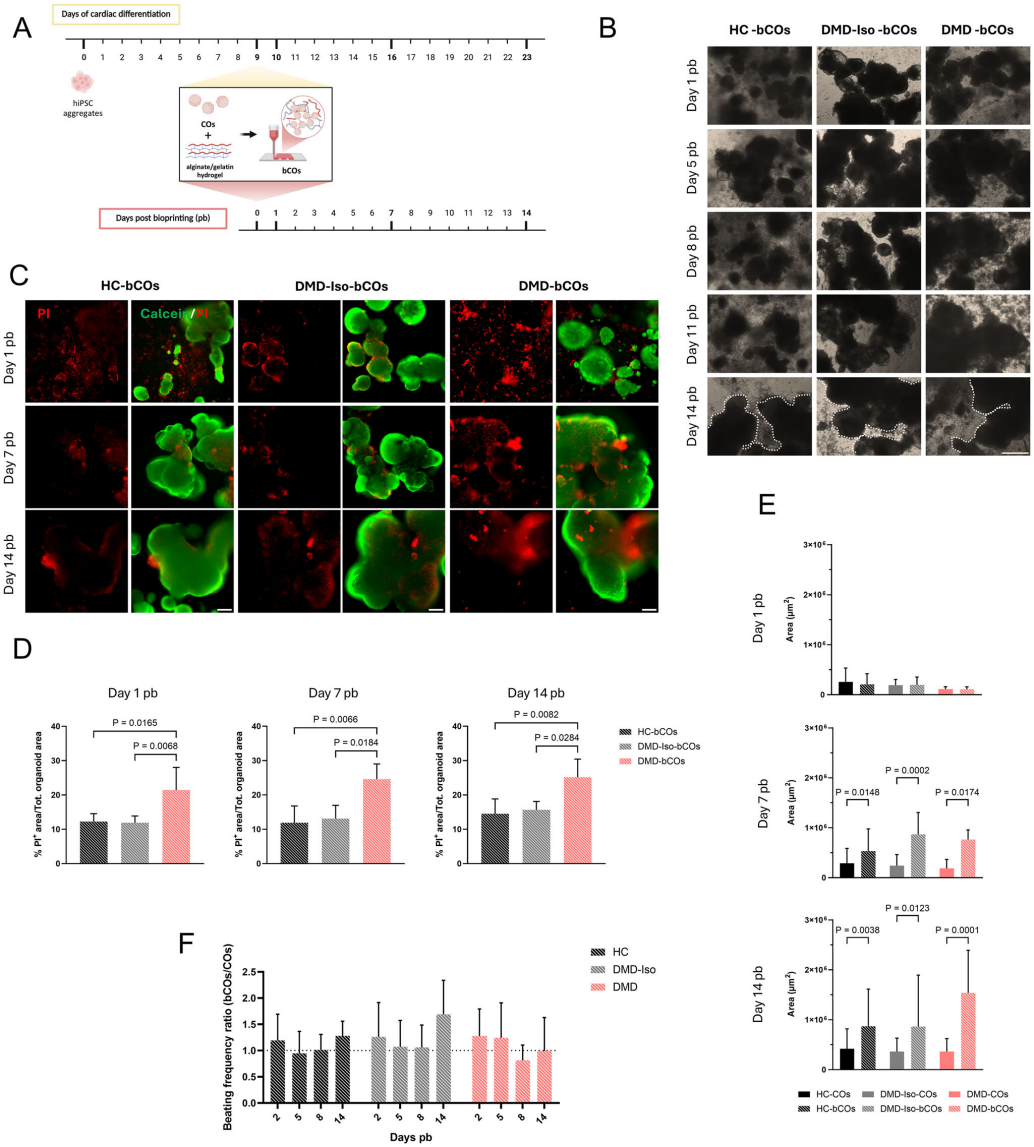
Generation of cardiac organoid‐based bioprinted constructs. (A) Schematic representation of the timeline of cardiac organoid differentiation and the bioprinting process. (B) Representative brightfield images showing the morphology of HC‐, DMD‐Iso‐, and DMD‐bioprinted COs (bCOs) at days 1, 5, 8, 11, and 14 post‐bioprinting (pb). Scale bar = 500 µm. (C) Representative live/dead staining images of HC‐, DMD‐Iso‐, and DMD‐bCOs at days 1, 7, and 14 pb. Calcein^+^ cells (green) = living cells; Propidium iodide^+^ cells (red) = dead cells). Scale bar = 250 µm. (D) Quantification of cell death as a percentage of PI^+^ area/ total organoid area in HC‐, DMD‐Iso‐, and DMD‐bCOs at days 1, 7, and 14 post‐bioprinting. Data are representative of at least three independent experiments (*n* ≥ 3) and expressed as mean ± SD. Statistical analysis was performed using one‐way ANOVA with Tukey's multiple comparisons test. (E) Quantification of CO and bCO areas at days 1, 7, and 14 post‐bioprinting. Data are representative of four or more independent experiments (*n* ≥ 4) and expressed as mean ± SD. Statistical analysis was performed using one‐way ANOVA with Šídák's multiple comparisons test. (F) Beating frequency ratio between bCOs and COs over 14 days post‐bioprinting. Data are representative of four or more independent experiments (*n* ≥ 4) and expressed as mean ± SD. Statistical analysis was performed using two‐way ANOVA with Tukey's multiple comparisons test between values from samples at the same time point.

HC‐COs embedded in a 3% alginate–2% gelatin remained viable and retained fusion capacity over time (Figure S5A), but its viscosity was insufficient for bioprinting. Thus, the concentrations of alginate and gelatin were increased to 7% and 5%, respectively (Figure 4). A schematic representation of the bioink preparation and bioprinting process is shown in Figure 4A. To assess morphological changes within the bioprinted COs (bCOs) compared to non‐embedded COs, brightfield images were captured at day 1, 5, 8, 11, and 14 post‐bioprinting (pb), (Figure 4B; Figures S5B,S6A). To evaluate cell viability, we performed live/dead staining at day 1, 7, and 14 pb in dystrophic, isogenic, and healthy bCOs, confirming the suitability of the hydrogel for long‐term culture (Figure 4C; Figure S6B). Notably, PI signal area, indicating cell death, was lowest in bioprinted HC‐COs (HC‐bCOs), followed by intermediate levels in DMD‐Iso‐bCOs, and highest in DMD‐bCOs (Figure 4D; Figure S6B). This trend became more pronounced over time, suggesting progressive viability loss in the dystrophic environment. Non‐bioprinted COs at days 1, 7, and 14 pb (Figure S5C,D) exhibited similar trends, further validating the hydrogel's role in supporting organoid survival. Both brightfield and live/dead images indicated a tendency of organoids to fuse within the hydrogel over time. Organoid area quantification confirmed this assumption, showing a significant increase in bCOs in comparison to COs from day 1 to day 14 pb across all cell lines (Figure 4E; Figure S5C). As a measure of function and cell viability, we also quantified beating frequency in these organoids. Average beating frequency values were used to calculate the ratio between bCOs and COs. Although not statistically significant, beating frequency in HC‐ and DMD‐Iso‐bCOs appeared to increase slightly over time. In contrast, dystrophic bCOs, exhibited an opposite trend (Figure 4F). These findings demonstrate that the gelatin–alginate hydrogel effectively supports organoid fusion and functional maintenance, allowing for the formation of larger, more complex structures.

### 2.5 Molecular Characterization of bCOs Reveals Temporal Gene Expression Changes and Protein Level Alterations in Healthy and Dystrophic Constructs

**FIGURE 5:**
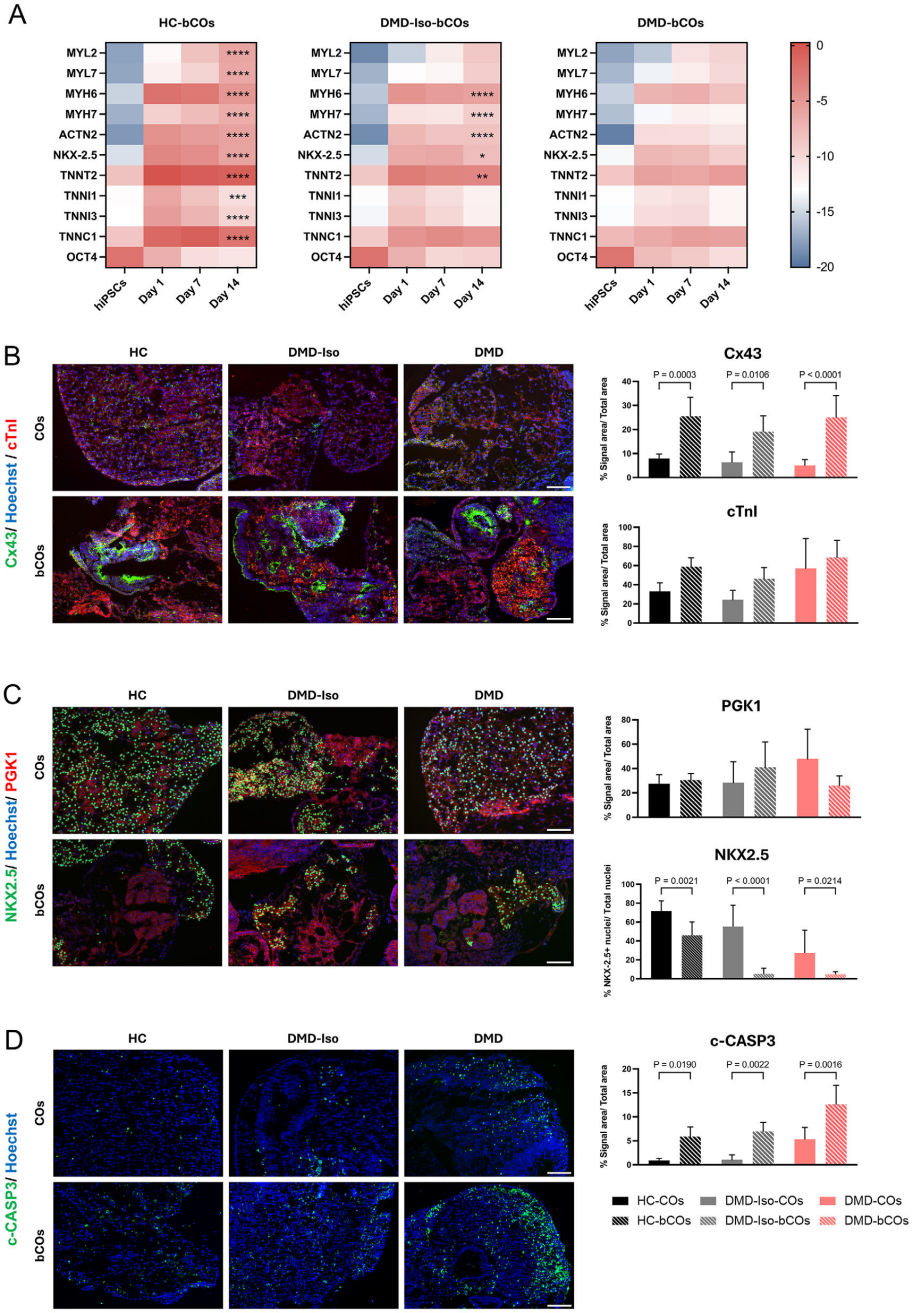
Assessment of cardiac differentiation, metabolic state, and apoptosis in HC, DMD‐Iso‐, and DMD‐bCOs. (A) Gene expression analysis of cardiac and pluripotency markers in HC‐, DMD‐Iso‐, and DMD‐bCOs at days 1, 7, and 14 post‐bioprinting and their respective hiPSC lines. Data are representative of four independent experiments (*n* = 4, each pooled of ∼50 organoids) and expressed as mean ± SD. Statistical analysis was performed using two‐way ANOVA with Dunnett's multiple comparisons test for day 14 post‐bioprinting: *p < 0.05, **p < 0.01, ***p < 0.001, ****p < 0.0001 indicate differences against DMD‐COs. (B–D) Representative immunofluorescence images (right panel) and quantification (left panel) of HC‐, DMD‐Iso‐, and DMD‐bCOs (*n* ≥ 4) for: B) cTnT/Cx43, C) PGK1/NKX2, D) CCASP3 at day 14 pb. Nuclei were counterstained with Hoechst. Data are representative of three independent experiments (*n* = 3). Statistical analysis was performed using one‐way ANOVA with Šídák's multiple comparisons test. Scale bar = 100 µm.

To assess the differentiation status of cardiac organoid‐based bioprinted constructs, we analyzed gene expression profiles at days 1, 7, and 14 pb (Figure 5). All cell lines showed a progressive upregulation of cardiomyocyte marker gene expression over time, while the expression levels of *OCT4* (pluripotency marker) decreased (Figure 5A; Figure S6D). When comparing gene expressions between constructs from different cell lines at day 14 pb, we observed a general downregulation of most cardiac markers (*MYH6*, *MYH7*, *ACTN2*, *TNNT2*, and *NKX‐2.5*) in DMD‐bCOs compared to DMD‐Iso‐ and HC‐bCOs. Notably, *MYL2*, *MYL7, TNNC1*, *TNNI1*, and *TNNI3* were significantly reduced in dystrophic constructs compared to HC‐bCOs. These findings indicate that while bioprinted constructs exhibit progressive CM differentiation, DMD‐bCOs show impaired cardiomyocyte marker expression, reinforcing the molecular deficits associated with DMD.

Immunofluorescence staining for cardiomyocyte, glycolytic, and apoptotic markers was conducted on healthy, isogenic, and dystrophic bioprinted constructs 14 days pb, with non‐embedded cardiac organoids serving as controls (Figure 5B–D). Localization of the cardiomyocyte gap junction protein Connexin 43 (Cx43) was increased in all bioprinted constructs compared to non‐embedded organoids, indicating enhanced cellular connectivity. Nevertheless, Cardiac Troponin I levels were comparable between bioprinted and non‐bioprinted organoids, suggesting consistent cardiac differentiation across all conditions (Figure 5B). Similarly, Phosphoglycerate Kinase 1 (PGK1), a key glycolytic marker, was equivalently distributed in all samples, regardless of the cell line. Conversely, NK2 Homebox 5 (NKX‐2.5), an early cardiac transcription factor associated with immaturity, was lower in all bioprinted constructs (Figure 5C). In the dystrophic bCOs, there was a notable increase in Cleaved Caspase 3 (CCASP3) levels, indicating a higher degree of apoptosis compared to bioprinted controls, and a more pronounced apoptotic phenotype than the non‐embedded organoids (Figure 5D). These results demonstrate that bioprinting promotes cardiac maturation while revealing increased cell death in a dystrophic environment, highlighting the vulnerability of dystrophic constructs to apoptosis.

### 2.6 Enhanced Extracellular Matrix Deposition in Dystrophic Bioprinted Constructs

**FIGURE 6:**
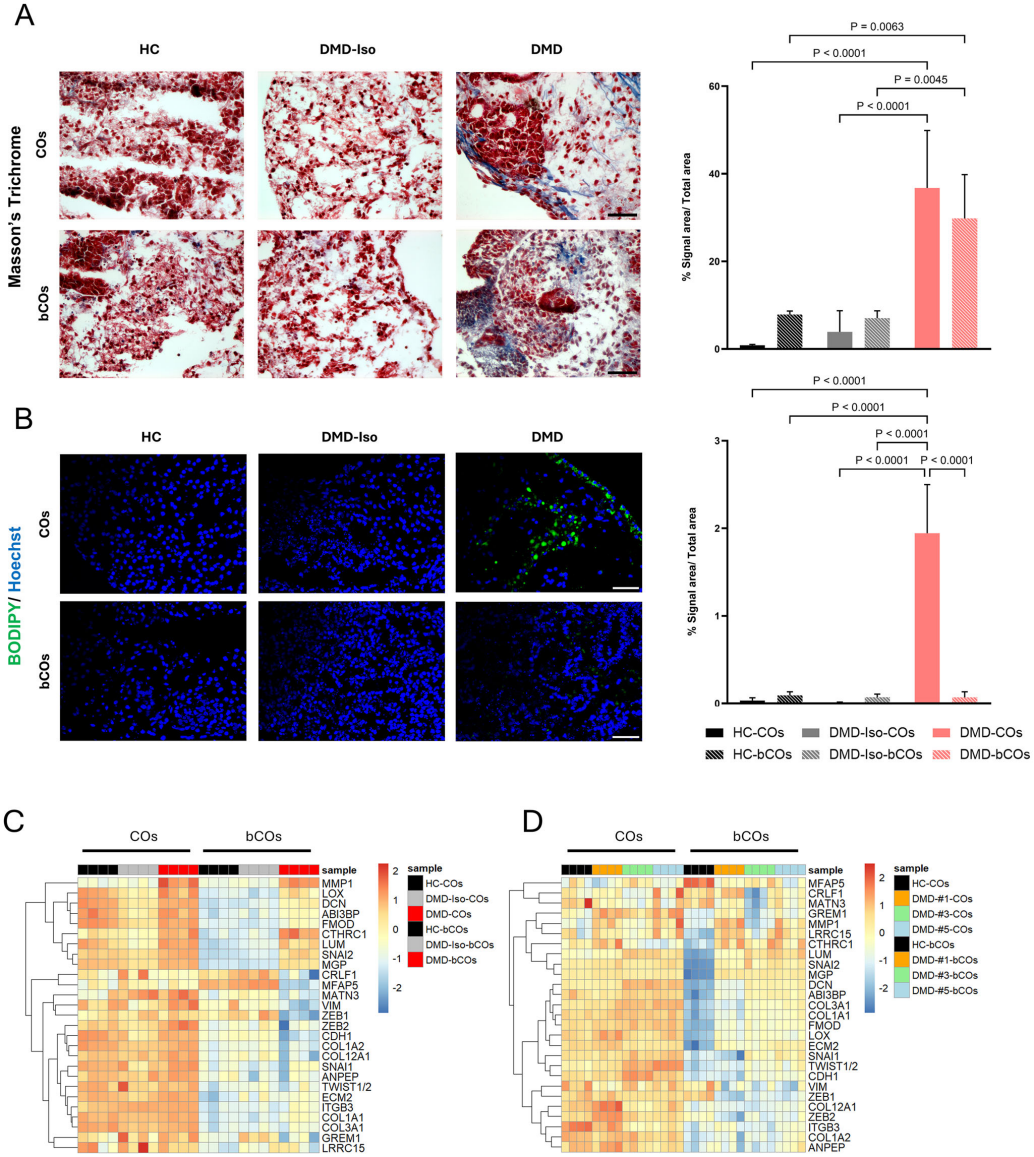
Assessment of fibrosis and adipogenesis in HC, DMD‐Iso, and DMD‐bCOs at day 14 pb. (A) Representative Masson's Trichrome staining (left panel) and quantification (right panel) showing collagen deposition in HC‐, DMD‐Iso‐, and DMD‐COs and bCOs at day 14 pb. (B) Representative BODIPY staining (left panel) and quantification (right panel) in HC‐, DMD‐Iso‐, and DMD‐COs and bCOs at day 14 pb. Data are representative of three independent experiments (*n* = 3) and expressed as mean ± SD. Statistical analysis was performed using one‐way ANOVA with Tukey's multiple comparisons; Scale bar = 50 µm. (C) Gene expression analysis of mesenchymal drift markers in HC‐, DMD‐Iso‐, and DMD‐COs and bCOs at day 14 post‐bioprinting. Data are representative of three independent experiments (*n* = 3, each pooled of ∼50 organoids). (D) Gene expression analysis of mesenchymal drift markers in HC‐, DMD #1‐, DMD #3‐, and DMD #5‐COs and bCOs at day 14 post bioprinting. Data are representative of three independent experiments (*n* = 3, each pooled of ∼50 organoids).

Two of the hallmarks of DMD‐associated cardiomyopathy include collagen fiber deposition and adipogenesis (Figure 6). Masson's trichrome staining analysis revealed a significant presence of collagen fibers in both DMD‐COs and DMD‐bCOs at day 14 pb, whereas DMD‐Iso and HC showed minimal to no collagen deposition regardless of bioprinting (Figure 6A). This suggests that the dystrophic environment actively promotes fibrosis, a hallmark of DMD‐associated cardiomyopathy. We then performed BODIPY staining, where we observed an increased lipid droplets accumulation in DMD‐COs compared with control COs (Figure 6B). However, this increased presence in fat deposition was not detected in DMD‐bCOs, suggesting that adipogenesis might have been influenced by the bioprinted environment.

To determine whether age‐related transcriptional changes described as *mesenchymal drift* are also present in DMD‐cardiomyopathy, we analyzed bulk RNA‐seq data comparing DMD‐ and DMD‐Iso‐COs at day 56 of differentiation (GSE194297). Of the 205 genes included in the MD signature, 147 exhibited dysregulated expression in DMD‐COs (Figure S7A). Next, we selected the top 28 DEGs in our day 56 DMD‐COs and analyzed their expression in dystrophic and healthy COs and bCOs on day 14 pb. Heatmap visualization revealed no apparent differences in expression between healthy and dystrophic COs; however, bCOs displayed a distinct separation (Figure 6C,D).

These findings confirm that dystrophic bioprinted constructs successfully recapitulate fibrosis‐associated pathological changes in DMD. This model provides a valuable 3D platform to study disease mechanisms and evaluate potential anti‐fibrotic interventions.

## 3 Discussion

Our study demonstrates the potential of 3D cardiac organoids and bioprinted cardiac constructs as relevant models to study DMD‐associated cardiomyopathy, highlighting key pathological features, including elevated oxidative stress, calcium dysregulation, increased apoptosis and cell death, and fibrosis. These findings provide valuable insights into disease mechanisms while offering a robust platform for therapeutic screening [26, 27].

Live/dead staining and flow cytometry analysis performed on COs confirmed a significantly higher proportion of dead and apoptotic cardiomyocytes in DMD cardiac organoids (DMD‐COs) compared to healthy and isogenic controls. These results are consistent with previous studies reporting elevated apoptosis in DMD cardiomyocytes, although the precise contribution of DMD mutations to programmed cell death pathways remains to be further elucidated [28, 29].

Notably, the increased cardiomyocyte apoptosis and death observed in DMD organoids were accompanied by tissue remodeling and greater stiffness. In contrast, healthy and isogenic controls exhibited comparable mechanical properties, suggesting that the increased stiffness in DMD organoids is linked to dystrophic cardiomyocytes. This observation is consistent with findings from 2D‐cultured DMD hiPSC‐derived cardiomyocytes, where elevated stiffness correlated with contractile dysfunction and oxidative stress, underscoring the role of mechanical stress in DMD progression [30]. Increased myocardial stiffness is also a hallmark of cardiac fibrosis, where tissue stiffness can rise two‐ to fourfold due to excessive accumulation of ECM components and the aberrant transformation of cardiac fibroblasts [31]. This was corroborated in our DMD model by the presence of fibrotic‐like tissue within the DMD‐COs. In our previous study, we detected a small population of PDGFRα^+^ cells (a marker for fibroblast progenitors) that might, under dystrophic conditions, differentiate into cardiac myofibroblasts (α‐SMA^+^) and drive their overactivation [6, 32].

Additionally, DMD‐COs displayed higher ROS accumulation accompanied by elevated *NOX4* expression and NADPH levels. Despite the cardioprotective effect of NOX4, its overexpression in the myocardium promotes cardiac remodeling through AKT‐mTOR and NFκB signaling, while its inhibition increases the cardiomyocyte contractility [33].

Given our previous identification of NOX4 as a key driver of oxidative stress‐induced apoptosis in dystrophic cardiomyocytes [14], its upregulation in DMD‐COs reinforces its central role in DMD‐related cardiomyocyte dysfunction.

Perturbations in excitation–contraction coupling (ECC) and Ca^2^^+^ handling are hallmark features of the remodeling DMD heart, which develops hypertrophic and dilated cardiomyopathies [34]. Aberrant Ca^2^^+^ transients and increased beating frequency observed in DMD‐COs indicate marked Ca^2^^+^ handling dysregulation, a well‐documented consequence of dystrophin deficiency in cardiomyocytes [35, 36, 37]. Loss of full‐length dystrophin compromises sarcolemmal integrity, promoting microtear formation that triggers the activation of L‐type Ca^2^^+^ channels and subsequent Ca^2^^+^ leakage from the sarcoplasmic reticulum (SR) [36]. This is supported by the dysregulation of several cardiac action potential channel genes (*SCN5A*, *KCNA5*, *KCNQ1*, *KCNJ2*, and *KCNJ12*) in dystrophic COs, possibly reflecting a compensatory mechanism to limit spontaneous Ca^2^^+^ waves through downregulation of sodium channels and upregulation of potassium channels.

Consequently, Ca^2^^+^ handling becomes severely impaired, as seen by the altered transients and the upregulation of multiple Ca^2^^+^ transporters and channels. Dystrophin deficiency further disrupts SR function and Ca^2^^+^ clearance, leading to sustained cytosolic Ca^2^^+^ elevation that exacerbates disease progression [36]. The prolonged time to peak and decay of Ca^2^^+^ transients in DMD‐COs likely reflects increased Ca^2^^+^ release through RYR2 and inefficient cytoplasmic clearance during diastole via SERCA and NCX, all upregulated in the dystrophic condition. These findings align with previous reports showing that dystrophin loss impairs cardiomyocyte maturation and contraction, resulting in reduced myofibrillar tension and abnormal Ca^2^^+^ dynamics [35]. Moreover, the re‐expression of fetal cardiac genes such as *NPPA, ITPR1, ITPR2*, *ITPR3*, and *CACNA1G* in DMD‐COs further supports ongoing cardiac remodeling, potentially amplifying contractile dysfunction and oxidative stress [34].

In this context, excessive NADPH oxidase (NOX)‐derived oxidative stress interferes with Ca^2+^ homeostasis in dystrophic cardiomyocytes. Indeed, NOX overactivation has been shown to interfere with SR function, while NOX inhibition was reported to partially restore Ca^2^^+^ handling and reduce arrhythmogenic events [37]. However, how NOX‐driven oxidative stress mechanistically intersects with SR dysfunction and Ca^2^^+^ dysregulation in DMD‐COs remains incompletely understood, warranting further investigation.

To scale up our model, we exploited bottom–up tissue engineering via 3D bioprinting. By combining 7% alginate and 5% gelatin with COs, we developed a bioink that mimics the native ECM and supports cardiomyocyte adhesion. This approach enhanced standardization and automation, allowing the reproducible fabrication of 3D cardiac constructs that preserve structural integrity after printing as well as after organoid integration.

The bioprinted constructs (bCOs) exhibited higher Connexin 43 (Cx43), a crucial component of the cardiomyocyte gap junctions, and lower NK2 Homeobox 5 (NKX2.5) levels, suggesting enhanced cardiomyocyte organization and intercellular coupling compared to non‐bioprinted organoids. This aligns with studies showing that 3D bioprinting promotes cardiomyocyte alignment, tissue organization, and gap junction formation, enhancing electrical conductivity and contractility [18].

Conversely, dystrophic bioprinted constructs (DMD‐bCOs) showed significantly reduced expression of structural genes (myosins, troponins, *ACTN2*, *NKX2.5*) compared to healthy control bCOs (HC‐bCOs). Interestingly, *MYL2*, *MYL7, TNNC1*, *TNNI1*, and *TNNI3* levels were comparable between DMD‐bCOs and isogenic controls (DMD‐Iso‐bCOs), indicating that their regulation may be influenced by the shared genetic background.

Notably, DMD‐bCOs also exhibited more apoptosis than non‐bioprinted DMD organoids, suggesting that enhanced cell–matrix and cell–cell interactions within the bioprinted constructs may intensify stress responses [38]. This aligns with findings demonstrating that higher tissue compaction and mechanical forces in bioprinted cardiac patches can trigger apoptosis and fibrosis in disease models [39].

Additionally, while bioprinting improves structural organization and functional properties, it introduces new challenges such as mechanical stress and limited vascularization leading to the development of hypoxic regions, potentially causing additional cell death in dystrophic contexts [18, 40].

Incorporating vascular networks into bioprinted constructs will be essential to achieve physiological oxygenation and nutrient exchange, addressing both scalability and disease complexity [41, 42]. Nonetheless, limitations such as immature contractile properties and diffusion constraints persist, highlighting the need for optimized bioink formulations, microenvironmental tuning, and potential co‐culture with cardiac fibroblasts and endothelial cells to enhance maturation [39, 43, 44].

Masson's trichrome staining revealed collagen deposition exclusively in dystrophic constructs, confirming the presence of fibrotic regions within the bioprinted environment. While fibrosis is a DMD hallmark, the role of cell–cell interactions and ECM remodeling in engineered cardiac tissues remains poorly understood. Bioprinting approaches incorporating self‐healing hydrogels have been shown to improve tissue fusion and ECM reorganization, which may affect fibrotic remodeling [45].

Additionally, prolonged culture may exacerbate matrix alterations, influencing both the fibrosis and the mechanical properties of the constructs. Hydrogel stiffness itself likely modulates fibrotic responses and even other pathological features such as aberrant adipogenesis in dystrophic organoids. Studies using engineered 3D cardiac spheroids under hypoxic conditions or cardiotoxic drugs have shown that ECM remodeling dynamically regulates fibrosis and cellular viability [46].

Moreover, fibroblast–cardiomyocyte interactions in bioprinted constructs can trigger excessive collagen deposition and impaired contractility, mimicking post‐infarct cardiac scarring [45].

In this broader context of remodeling and fibrosis, recent work by Lu et al. has described mesenchymal drift as a stress‐associated process contributing to fibrotic progression across multiple disease settings [11]. Guided by this framework, we explored whether signatures associated with mesenchymal drift are also detectable in our DMD‐CO models. Notably, DMD bioprinted cardiac organoids (DMD‐bCOs) showed an enrichment of mesenchymal drift‐associated gene expression compared to healthy controls, including markers linked to mesenchymal activation and remodeling. While exploratory, these findings suggest that the bioprinted environment, through enhanced mechanical constraints, ECM interactions, may promote lineage‐related stress responses previously described in other fibrotic diseases, extending their relevance to a DMD‐specific cardiac setting.

These results emphasize the need for optimized biofabrication strategies that balance ECM remodeling while preserving functional cardiac tissue properties in DMD models. Taken together, our results underscore the complex interplay between bioprinting, ECM dynamics, and fibrosis in dystrophic constructs.

## 4 Conclusion

Our study demonstrates that 3D cardiac organoids and bioprinted cardiac constructs provide a robust and reproducible platform to model DMD‐associated cardiomyopathy, recapitulating key pathological features such as enhanced apoptosis, oxidative stress, calcium dysregulation, and ECM remodeling associated with fibrosis. By integrating organoid self‐organization with controlled bioprinting, this model achieves improved structural consistency and scalability, enabling more consistent detection of disease‐associated phenotypes. The upregulation of NOX4 and the presence of altered Ca^2^^+^ transients within dystrophic constructs highlight the interplay between oxidative stress, sarcoplasmic reticulum dysfunction, and apoptosis, implicated in DMD cardiomyopathy, and demonstrate that these features are captured in our 3D human models. The bioprinted microenvironment appears to modulate tissue organization and stress responses, in some cases accentuating disease‐related phenotypes in dystrophic constructs. These observations underscore the influence of matrix composition and mechanical context on the manifestation of DMD‐associated pathology.

A key limitation of our current model is the incomplete representation of cardiac tissue complexity, including the absence of endocardial and epicardial cell layers and limited vascularization, which may restrict long‐term tissue viability and influence fibrotic remodeling. Future studies should focus on refining bioink composition, incorporating vascular and stromal cell populations, and controlling oxygen and mechanical gradients to further enhance pathophysiological relevance [45, 46].

Overall, our findings position standard and bioprinted cardiac organoids as complementary 3D platforms for comparative disease modeling and preclinical investigation in DMD, bridging the gap between reductionist 2D models and in vivo systems, and providing a foundation for future preclinical studies and therapeutic screening efforts.

## 5 Experimental Section

### 5.1 Human Induced Pluripotent Stem Cell Lines

The cell lines used to model DMD‐associated cardiomyopathy were generated by reprogramming of dermal fibroblasts from a DMD donor patient (DMD‐hiPSCs,) carrying a nonsense point mutation in exon 35 (c.4 996C > *T*; p.Arg1,666X, DMD #2 [14]) leading to a premature stop codon. The DMD isogenic control line (DMD‐Iso‐hiPSCs) was generated by correction of this mutation through CRISPR/Cas9 [14]. The additional DMD #1‐ (deletion exon 49–52), DMD #3‐ (duplication exon 3–11), and DMD #5‐hiPSC (deletion exon 51–55) lines were generated from the PBMCs of three different donor DMD patients and reprogrammed toward pluripotency using the integration‐free Sendai virus (SeV)‐based technology. The healthy, control hiPSC line (HC‐hiPSCs) was generated through SeV‐based reprogramming of male donor fibroblasts as previously reported [14]. The use of human samples from DMD subjects for experimental purposes and protocols in the present study was approved by the Ethics Committee of the University Hospitals Leuven (S66794).

### 5.2 Maintenance and Expansion of hiPSCs

Human iPSC lines were cultured feeder‐free on Geltrex LDEV‐Free hESC‐qualified reduced growth factor basement membrane matrix. All hiPSCs were maintained in Essential 8 flex basal medium (Thermo Fisher Scientific) supplemented with Essential 8 Flex Supplement (50×, Thermo Fisher Scientific) and penicillin–streptomycin (0.1%, Thermo Fisher Scientific) at 37°C under standard tissue culture conditions (21% O_2_ and 5% CO_2_). Colonies were routinely passaged non‐enzymatically with 0.5 mm EDTA in phosphate‐buffered saline (PBS, Thermo Fisher Scientific). Harvesting of hiPSCs and single‐cell disaggregation for differentiation purposes was accomplished through incubation with Accutase solution (Sigma Aldrich) at 37°C for 15 min, and subsequent resuspension in 10% (v/v) Fetal Bovine Serum (FBS; Thermo Fisher Scientific) in E8 Medium.

### 5.3 Myocardial Differentiation

DMD‐, DMD #1‐, DMD #2‐, DMD #3‐, DMD‐Iso‐, and HC‐hiPSC lines were differentiated into functional cardiomyocytes with the use of the PSC cardiomyocyte differentiation kit (ThermoFisher Scientific). The kit includes 3 different ready‐to‐use serum‐free medium compositions: medium A (Wnt pathway activation), medium B (Wnt pathway inhibition), and cardiomyocyte maintenance medium (MM). The cells were maintained in E8 medium at 37°C under reduced oxygen conditions (5% O_2_ and 5% CO_2_) for 3 days (until 2D cultures reached 85% confluency). Induction of mesodermal differentiation was performed on day 0 by changing to medium A. After 24 h (day 1), the cells were transferred to another incubator and maintained at 21% O_2_ and 5% CO_2_ from then on. On day 2, cells were further pushed toward cardiac progenitors by administering Medium B. From day 4 onward, Cardiomyocyte MM was added and refreshed every two days until cells were collected for analysis. Contracting CMs started appearing at day 8–9 of cardiac differentiation.

### 5.4 hiPSC‐Derived Organoids From Agarose Molds

In brief, hiPSC‐derived cardiac organoids were created by culturing the hiPSCs in custom‐made agarose molds and applying the cardiac differentiation protocol. Agarose molds were generated with a custom‐made 3D printed micropillar plaque containing 24 inserts, each consisting of 137 microwells (diameter × height = 500 × 700 µm). A gel solution of 3% (w/v) agarose (Invitrogen) in PBS was prepared through thermal dissolution and was added to the micropillar plaques. After cooling down for 10 min at RT, the agarose molds were removed from the plaque, transferred to a 24‐well plate, and equilibrated in PBS overnight at 37°C. To generate hiPSC‐derived organoids, the cells were disaggregated into single cells by incubating 15 min with Accutase solution (Sigma Aldrich) at 37°C. Cells were then counted and resuspended in Essential 8 flex basal medium (Thermo Fisher Scientific) with RevitaCell supplement 1: 100 and plated into the agarose molds at a density of 1 × 10^4^ cells/microwell. Next, plates were centrifuged for 10 min at 200 g to ensure hiPSCs were settled at the bottom of the microwells. Subsequently, myocardial differentiation was performed following the previously described protocols. On day 5 of differentiation, organoids were removed from agarose molds and transferred to an ultra‐low attachment 6‐well plate (Costar, Corning) incubating on an orbital shaker at 75 rpm for dynamic culture conditions. Contracting COs started to appear from day 8 of the differentiation protocol. The samples were collected for further analysis at day 15, or at later timepoints as specified in the text (Table 1).

**TABLE 1:** Primary Antibodies for immunofluorescence staining.

Antibody	Host species	Catalog number	Dilution
Cardiac troponin I	Goat	Abcam (ab56357)	1:200
Cleaved caspase‐3	Rabbit	Cell signaling technology (9661S)	1:400
Connexin 43	Rabbit	Abcam (ab11370)	1:500
NKX2.5	Rabbit	Bioké (E1Y8H)	1:250
PGK1	Mouse	Santa cruz biotechnology (sc‐130335)	1:250
Cardiac troponin T	Rabbit	Abcam (ab45932)	1:600
Sarcomeric alfa actinin	Mouse	Abcam (ab9465)	1:100
Alfa‐smooth muscle actin	Mouse	Abcam (ab7817)	1:1000
Collagen I alpha 1	Rabbit	BioTechne (NP1‐30054)	1:250
PECAM1	Mouse	Agilent (M082329‐2)	1:100
Perilipin	Rabbit	Bioké (9349T)	1:200

### 5.5 Flow Cytometry Analysis

DMD‐, DMD‐Iso‐, and HC‐COs at 15 days were dissociated using Collagenase A (1 U/mL) for 20 min at 37°C followed by 10 min incubation with Accutase (Sigma‐Aldrich). All flow cytometry procedures were performed according to the manufacturer's instructions. PBS + 0.1% BSA (Sigma) was used as staining buffer. The cells were stained for the surface markers SIRPA‐PE specific for CMs, Annexin V‐APC specific for apoptotic cells. As viability dye DAPI and eFluor780 (Invitrogen) were used. A total of 50 000 cells was recorded. After single‐cell gate selection, a total cell number between 15 000 and 20 000 cells was analyzed for the used antibodies (SIRPA, Annexin V). Fluorescence minus one (FMO) controls, and compensations were included for appropriate gating. Samples were measured using the FACS Canto II HTS (BD Biosciences) and the analysis was performed using FACS Diva Software. Table 2 provides a list of all flow cytometric antibodies used in this study.

**TABLE 2:** List of primers used for gene expression analysis.

Gene name	Sequences
*NOX4*	Fw 5′‐TCCGGAGCAATAAGCCAGTC ‐3′ Rv 5′‐ CCATTCGGATTTCCATGACAT ‐3′
*NOX2*	Fw 5′‐ TGCCAGTCTGTCGAAATCTGC ‐3′ Rv 5′‐ ACTCGGGCATTCACACACC‐3′
*p22^phox^*	Fw 5′‐TACTATGTTCGGGCCGTCCT‐3′ Rv 5′‐CACAGCCGCCAGTAGGTA ‐3′
*P47^phox^*	Fw 5′‐ GGGGCGATCAATCCAGAGAAC ‐3′ Rv 5′‐ GTACTCGGTAAGTGTGCCCTG ‐3′
*RAC1*	Fw 5′‐ ATGTCCGTGCAAAGTGGTATC ‐3′ Rv 5′‐ CTCGGATCGCTTCGTCAAACA ‐3′
*RAC2*	Fw 5′‐ TCTGCTTCTCCCTCGTCAG ‐3′ Rv 5′‐ TCACCGAGTCAATCTCCTTGG ‐3′
*RAC3*	Fw 5′‐ CTTCGAGAATGTTCGTGCCAA ‐3′ Rv 5′‐ CCGCTCAATGGTGTCCTTG ‐3′
*ACTN2*	Fw 5′‐CTCAAGAGTCAAAGAGTATC‐3′ Rv 5′‐GTGGATGGAAGAAGAGCAGTA‐3′
*MYH6*	Fw 5′‐GCCCTTTTGACATTCGACTG‐3′ Rv 5′‐CGGGAAAAATTCGTGGTTTA‐3′
*MYH7*	Fw 5′‐ACTGGCAGAGTCACAGGTAAC‐3′ Rv 5′‐GCATCCTTCAGGTTGTTGAGAG‐3′
*MYL2*	Fw 5′‐TTGGGGGTAGTGAGTGGAAAA‐3′ Rv 5′‐CGGAAGCAATACAGCCTTAG‐3′
*MYL7*	Fw 5′‐ACATATACCCACCGGAAAGC‐3′ Rv 5′‐ATTGGAGATGGCCTTGGAGATGA‐3′
*NKX2.5*	Fw 5′‐ TTGTCATTCACCTCTGCGGAACCTA ‐3′ Rv 5′‐ TGCAGCCTGACTAATCGCG ‐3′
*OCT4*	Fw 5′‐ GAAGGATGTGGTCCGAGTG ‐3′ Rv 5′‐ GTGAGGGAAGCTGGAGAAAG ‐3′
*TNNC1*	Fw 5′‐ CAGGAGACCAAGAAGAAGC ‐3′ Rv 5′‐ CGATTCTGGAACACTTGGC ‐3′
*TNNI3*	Fw 5′‐ GGCGCTCCAGAGGAAATTA ‐3′ Rv 5′‐ AGAGTGAGTCCGAGGGAAC ‐3′
*TNNI1*	Fw 5′‐ CCCAGGACCAGGCTACTG ‐3′ Rv 5′‐ TGGAAGGTTGAAGGGGTAG ‐3′
*TNNT2*	Fw 5′‐ GAGGTCACAGGATAGTTCC ‐3′ Rv 5′‐ TCAAAGTCCACTTCTGTCATC ‐3′
*MMP1*	Fw 5′‐ GGGGCTTTGATGTACCCTAGC ‐3′ Rv 5′‐ TGTCACACGCTTTTGGGGTTT ‐3′
*LOX*	Fw 5′‐ ACCACAGGCGATTTGCATGTA ‐3′ Rv 5′‐ GGCAGTCTATGTCTGCACCA ‐3′
*DCN*	Fw 5′‐ ATGAAGGCCACTATCATCCTCC ‐3′ Rv 5′‐ GTCGCGGTCATCAGGAACTT ‐3′
*ABI3BP*	Fw 5′‐ CAAATGCAACATGCTCTCCAGT ‐3′ Rv 5′‐ TTGGCCTTTTACCTTTTGGCA ‐3′
*FMOD*	Fw 5′‐ GAGACCTACGAGCCTTACCC ‐3′ Rv 5′‐ TTGAGGTTGCGATTGTCACAG ‐3′
*CTHRC1*	Fw 5′‐ CAATGGCATTCCGGGTACAC ‐3′ Rv 5′‐ GTACACTCCGCAATTTTCCCAA ‐3′
*LUM*	Fw 5′‐ CTGCGTTTATCTCACAACGAACT ‐3′ Rv 5′‐ CAGATCCAGCTCAACCAGGG ‐3′
*SNAI2*	Fw 5′‐ TGCATATTCGGACCCACACA ‐3′ Rv 5′‐ TGTTGCAGTGAGGGCAAGAA ‐3′
*MGP*	Fw 5′‐ TCCGAGAACGCTCTAAGCCT ‐3′ Rv 5′‐ GCAAAGTCTGTAGTCATCACAGG ‐3′
*CRLF1*	Fw 5′‐ CTCTCCCGTGTACTCAACGC ‐3′ Rv 5′‐ GGGCAGGCCAACATAGAGG ‐3′
*MFAP5*	Fw 5′‐ ACACGAAGCTATGAAAGATGAGC ‐3′ Rv 5′‐ AGTCGGAAGTAATTGGAGCGA ‐3′
*MATN3*	Fw 5′‐ TCTCCCGGATAATCGACACTC ‐3′ Rv 5′‐ CAAGGGTGTGATTCGACCCA ‐3′
*VIM*	Fw 5′‐ TCCAAGTTTGCTGACCTCTCTG ‐3′ Rv 5′‐ CAGTGGACTCCTGCTTTGCC ‐3′
*ZEB1*	Fw 5′‐ GATCCAGCCAAATGGAAATCA ‐3′ Rv 5′‐ GGCGGTGTAGAATCAGAGTCATTC ‐3′
*ZEB2*	Fw 5′‐ GCGATGGTCATGCAGTCAG ‐3′ Rv 5′‐ CAGGTGGCAGGTCATTTTCTT ‐3′
*CDH1*	Fw 5′‐ GACACCAACGATAATCCTCCGA ‐3′ Rv 5′‐ GGCACCTGACCCTTGTACGT ‐3′
*COL1A2*	Fw 5′‐ GTTGCTGCTTGCAGTAACCTT ‐3′ Rv 5′‐ AGGGCCAAGTCCAACTCCTT ‐3′
*COL12A1*	Fw 5′‐ GGAGTGGAGCTGTTTGCTATT ‐3′ Rv 5′‐ GAGAGTGACTCAAAATCTGCCA ‐3′
*SNAI1*	Fw 5′‐ TCGGAAGCCTAACTACAGCGA ‐3′ Rv 5′‐ AGATGAGCATTGGCAGCGAG ‐3′
*ANPEP*	Fw 5′‐ TTCAACATCACGCTTATCCACC ‐3′ Rv 5′‐ AGTCGAACTCACTGACAATGAAG ‐3′
*TWIST1/2*	Fw 5′‐ GTCCGCAGTCTTACGAGGAG ‐3′ Rv 5′‐ GCTTGAGGGTCTGAATCTTGCT ‐3′
*ECM2*	Fw 5′‐ CCGAATGCCCTCTCGATCC ‐3′ Rv 5′‐ TGGGTAAGCATGGCGTTGATG ‐3′
*ITGB3*	Fw 5′‐ AGTAACCTGCGGATTGGCTTC ‐3′ Rv 5′‐ GTCACCTGGTCAGTTAGCGT ‐3′
*COL1A1*	Fw 5′‐ GAGGGCCAAGACGAAGACATC ‐3′ Rv 5′‐ CAGATCACGTCATCGCACAAC ‐3′
*COL3A1*	Fw 5′‐ TTGAAGGAGGATGTTCCCATCT ‐3′ Rv 5′‐ ACAGACACATATTTGGCATGGTT ‐3′
*GREM1*	Fw 5′‐ TCATCAACCGCTTCTGTTACG ‐3′ Rv 5′‐ GGCTGTAGTTCAGGGCAGTT ‐3′
*LRRC15*	Fw 5′‐ TATCTCAGCCTCGCCAACAAC ‐3′ Rv 5′‐ AGCTGGTTACTGGACAGAAGG ‐3′
*RPL13A*	Fw 5′‐ CCTGGAGGAGAAGAGGAAAGAGA ‐3′ Rv 5′‐ TTGAGGACCTCTGTGTATTTGTCAA ‐3′
*GAPDH*	Fw 5′‐ TGGACTCCACGACGTACTCA ‐3′ Rv 5′‐ TCAGCAATGCCTCCTGCACC ‐3′
*RPS9*	Fw 5′‐ CGGAGACCCTTCGAGAAATC ‐3′ Rv 5′‐ GGATCTTGGCCAGGGTAAAT ‐3′
*RPS15A*	Fw 5′‐ CAGTAACCTCAGCATCCGTATC ‐3′ Rv 5′‐ CCAAGTGCGCTTTACACAATC ‐3′
*HPRT*	Fw 5′‐ TGACACTGGCAAAACAATGCA ‐3′ Rv 5′‐ GGTCCTTTTCACCAGCAAGCT ‐3′

### 5.6 Mechanical Characterization

Mechanical experiments were performed using a MicroTester LT (CellScale) device. Cardiac organoids were tested alive in a cardiomyocyte maintenance medium (MM) bath at 37°C. Strain‐controlled compression experiments were performed using tungsten beams to compress the cardiac organoids to strains of 50%. Finally, an integrated camera with a 2× zoom objective was used to record the force deformation and size measurement of the spheroids.

### 5.7 Reactive Oxygen Species Production

At day 15, DMD‐, DMD‐Iso‐, and HC‐derived cardiac organoids (COs) were enzymatically dissociated using Collagenase A (1 U/mL) for 20 min at 37°C, followed by a 10‐minute incubation with Accutase (Sigma‐Aldrich) to obtain single‐cell suspensions. Intracellular ROS levels were quantified in live, unfixed cardiomyocytes using the total ROS assay kit (520 nm; ThermoFisher, 88‐5930‐74), following the manufacturer's protocol. The assay enables the detection of ROS via flow cytometry in the FITC channel. Cardiomyocytes were identified based on expression of SIRPA, detected using an APC‐conjugated antibody specific to this cardiomyocyte marker. A minimum of 5 × 10^4^ cellular events were acquired per sample using a FACS Canto II HTS system (BD Biosciences). Flow cytometry data were subsequently analyzed using FlowJo software (TreeStar, Ashland, OR).

### 5.8 Measurements of NADPH‐Dependent ROS Production

The Colorimetric NADPH Assay Kit (Abcam) provides a convenient method for detecting NADPH in contrast to the traditional NAD/NADH and NADP/NADPH assays, which monitor the changes in NADH or NADPH absorption at 340 nm [47]. Here, the NADPH probe is a chromogenic sensor that has its maximum absorbance at 460 nm upon NADPH reduction. The absorption of the NADPH probe is directly proportional to the concentration of NADPH. Briefly, COs (20 000 cells per well in 100 µL volume) were seeded in a 96‐well black microplate with clear flat bottoms. The NADPH probe was added to the samples and incubated for 30 min protected from light. Recordings were performed with an ELx808 Absorbance Microplate Reader with absorbance measurements set at 460 nm and quantified using Gen5 Software Version 3 (both from BioTek Instruments).

### 5.9 Bioink Synthesis

Gelatin from porcine skin (MP Biomedicals LLC, VWR) was dissolved in 10 mL of cardiomyocyte maintenance medium (MM) at a final concentration of 5% (w/v) and mixed with low viscosity Alginic acid sodium salt powder (MP Biomedicals LLC, Bio‐connect) to reach the final concentration of 7% (w/v). Following the synthesis, the 5% Gelatin/ 7% Alginic acid solution was incubated at 37°C overnight to fully hydrate, under shaking conditions.

### 5.10 Post‐Printing Accuracy and Resolution

The bioprinting procedure was evaluated for its accuracy and resolution. The volumetric extrusion was set at 60 and 4 mm/s speed for the extrusion and substrate, respectively via Slic3r settings. The line width analysis was carried out by printing the demonstrated diluted condition, in a 3D construct, and taking multiple measurements regarding the width of the layers. Additionally, 3D cylindrical constructs with dimensions of 6 mm in diameter and 2 mm in height were printed and analyzed for their final diameter.

### 5.11 Rheological Analysis

Rheological characterization was performed with an MCR702 (Anton Paar) utilizing a 15 mm parallel plate configuration. The geometric gap was maintained constant throughout the measurements of the bioink. Initially, an oscillatory strain sweep was performed over a range of 0.01%–100% with an oscillation frequency of 1 Hz to identify the linear viscoelastic region (LVR). Next, a frequency sweep of oscillations between 0.01 and 100 Hz was used to monitor variations in the storage modulus (G′) and the loss modulus (G″) at a constant strain in the linear viscoelastic range (LVR). Finally, viscosity tests were conducted by an incremental increase in shear rate from 0.01 to 100 s^−1^.

### 5.12 Hydrogel‐Embedded 3D Myocardial Construct Generation

COs were printed on day 9 of differentiation using a 7% alginic acid–5% gelatin hydrogel at a density of 11 × 10^3^ organoids/mL. The cardiac structures were printed with a custom‐made bioprinter, modified from a low‐cost commercial Ender 3 printer. The printer was controlled by the Printrun Software through a connected computer. The computer‐aided design program, named SketchUp (Trimble Inc.) designed the 3D printing model with dimensions of 6 mm × 6 mm × 8 mm. Printing was performed at RT, using a 5 mL Luer Lock syringe with an 18G needle (internal diameter of 0.84 mm) that was placed in the 3D printer. The volumetric extrusion was set at 60 and 4 mm/s speed for the extrusion and substrate, respectively via Slic3r settings. By converting the rotational stepper motor's torque to linear force, the pressure at the tip of the needle was calculated. The rate of bioink extrusion was estimated at 0.0008 mL/s as the mechanical extrusion velocity of the driven piston was approximately 0.0073 mm/s. According to Bernoulli's principle and the continuity equation, considering the inner 18G needle radius of 0.42 mm and the inner 5 mL syringe radius of 6.035 mm, a pressure of 60.78 kPa was calculated. All bioprinted constructs were cross‐linked with 2% CaCl_2_ in MilliQ for 3 min, washed twice with MM, and transferred into a non‐adherent 24‐well suspension plate (Costar, Corning). The 3D myocardial constructs were further maintained in MM under standard tissue culture conditions. Samples for immunostaining were collected on day 14 after bioprinting, fixed with 4% PFA, and embedded in cryogel. Samples for RT‐qPCR were collected on days 1, 7, and 14 post‐bioprinting.

### 5.13 Cell Culture Live‐Imaging

Live imaging of cells was performed using a Leica DMi microscope (Leica Microsystems). Images and videos were obtained through the related Leica application suite EZ v3.4.0 (Leica Microsystems) software.5.1

### 5.14 Quantification of Embedded Organoids Fusion and Beating Frequency

To assess the contractile properties of DMD‐COs, DMD‐Iso‐ and HC‐bCOs, 3D cardiac organoids were live‐imaged under brightfield using the DMI1 microscope (Leica). The recorded videos were then analyzed to determine the CO beating frequency by counting the number of spontaneous contractions per minute. The beating frequency ratio was calculated by dividing the beating frequency values of bCOs by their control CO counterparts. The cardiac organoid growth area was measured at different time points using ImageJ [48].

### 5.15 Calcein and Propidium Live/Dead Staining

To determine the cell viability within the COs and the bCOs, a live/dead cell staining analysis was performed. To this end, the constructs incubated in MM were supplemented with Calcein‐AM (dilution 1:2000) and propidium iodide (dilution 1:1000) for 1 h (LIVE/DEAD viability/cytotoxicity kit, for mammalian cells, Thermofisher). Imaging was done with the Nikon Eclipse Ti microscope. Quantification was performed using ImageJ software by calculating the percentage of propidium iodide‐positive area with respect to the total organoid area in each image.

### 5.16 Cryogel‐Embedded Cardiac Tissue Sectioning

Multiple 3D COs and bioprinted constructs were collected per condition and fixed in 4% (w/v) paraformaldehyde (PFA; Polysciences) in PBS for 30 min at RT. Following a PBS washing step, the constructs were transferred to a cassette and filled with Tissue‐Tek O.C.T. Compound (Sakura Finetek USA). Samples were flash‐frozen in liquid nitrogen and stored at −80°C. Sectioning was performed using the HM525 NX Cryostat (Thermo Fisher Scientific) to obtain sections with a thickness of 8 µm. The resulting slides were stored at −80°C until further analysis.

### 5.17 Immunofluorescence Staining

Slides containing cryosections were immersed in PBS for 5 min followed by permeabilization in 0.1% (v/v) Triton X‐100 in PBS for 1 h at RT. To prevent nonspecific antibody binding, samples were blocked by incubation for 30 min at RT in PBS containing 10% normal donkey serum (Dako, Agilent Technologies) and 1% (w/v) bovine serum albumin (BSA; Sigma Aldrich). Slides were then incubated with primary antibodies (Table 1) diluted in PBS/1% BSA (w/v) at 4°C overnight. On the next day, sections were washed 3× in PBS for 5 min each on a shaker at 15 rpm, followed by a 30 min wash with 0.1% (v/v) Triton X‐100 in PBS. Samples were incubated in the dark with the equivalent AlexaFluor‐conjugated secondary antibodies (Invitrogen, Thermo Fisher Scientific) diluted 1:500 in PBS + 1% (w/v) BSA for 1 h. After three more washes with PBS, nuclei were stained with Hoechst 33342 diluted 1:1000 in PBS/1% (w/v) BSA through a 7 min incubation at RT. Sections were washed twice with PBS prior to mounting with ProLong Gold antifade reagent (Invitrogen). Samples were stored at 4°C in the dark. The Zeiss AxioImager Z1 epifluorescence microscope (Carl Zeiss) with AxioVision SE64 Rel. 4.9.1 (Carl Zeiss) software was employed for all imaging. ImageJ was used for further image processing. Quantification of immunofluorescence images was performed using ImageJ software by calculating either the percentage of positive signal area relative to the total organoid area or the percentage of positive nuclei relative to the total number of nuclei, depending on the marker analyzed.

### 5.18 Masson's Trichrome and BODIPY Staining

The presence of fibrotic depositions in bioprinted and control constructs was assessed by Masson's Trichrome staining (25 088, Polysciences). In short, slides were treated for 1 h with pre‐warmed Bouin's Fixative at 35°C, followed by a (1) 10 min incubation in Weigert's Iron Hematoxylin, (2) 5 min in Biebrich Scarlet – Acid Fuchsin Solution, (3) 10 min in phospotungstic/phosphomolybdic acid, (4) 5 min in Aniline Blue solution, and (5) 1 min in 1% acetic acid. Slides were rinsed in distilled water between each of these steps. Finally, sections underwent dehydration through sequential incubations in 95% and 100% Ethanol, prior to a final incubation in 100% Xylene before mounting the slides with DPX mountant (Sigma‐Aldrich).

BODIPY staining was used to detect lipid droplet deposition. Slides were stained with BODIPY 493/503 4,4‐Difluoro‐1,3,5,7,8‐Pentamethyl‐4‐Bora‐3a,4a‐Diaza‐s‐Indacene (Invitrogen) as previously described (Marini et al., 2022). Next, sections were incubated with Hoechst 33 342 diluted 1: 1000 in 1% (w/v) BSA in PBS for 7 min at RT for nuclei staining, which was followed by two PBS washes before mounting the slides with ProLong Gold antifade reagent (Invitrogen). Imaging was performed using the Zeiss AxioImager Z1 epifluorescence microscope (Carl Zeiss) with AxioVision SE64 Rel. 4.9.1 (Carl Zeiss) software. Further image processing and quantification were performed using ImageJ. Quantification of fibrotic depositions was performed by calculating the percentage of blue‐stained surface in Masson's Trichrome‐stained slides with respect to the total organoid area, whereas quantification of lipid droplets was assessed by calculating the percentage of BODIPY+ signal area relative to the total CO/bCO area.

### 5.19 Calcium Transient Assessment

For the purpose of imaging of calcium dynamics, beating COs were plated one by one into 96‐well SensoPlate glass‐bottom microplates (Greiner Bio‐One). Intracellular Ca^2+^ levels were monitored using Cal‐520, which was loaded into cells in its AM ester form (Cal520 AM, AAT Bioquest)). To this end, cells were loaded at 37°C for 1 h in MM containing 4 µm Cal‐520 AM and 0.04% Pluronic F‐127 (Thermo Fisher Scientific). De‐esterification was achieved by exchanging the dye‐containing medium with fresh MM. Recordings of Ca^2+^ dynamics were obtained using an FDSS/µCELL system (Hamamatsu Photonics). Ca^2+^ imaging was performed at 37°C under 0.5 Hz, and 1 Hz stimulation. The time course of fluorescence signal intensity changes per well (*F*) was determined using the FDSS software. Data were normalized by dividing each individual *F‐*value by the lowest *F*‐value of the corresponding organoid within each stimulation time frame (*F*_0_). The Waveform analysis software, included in the FDSS/µCELL system, was used to analyze raw intensity values to establish frequency, time between peaks (P–P time), peak amplitude, area under the curve (AUC), and rising and falling slopes. Double peaks were defined as the presence of two distinct contraction maxima within a single electrical stimulation interval and were manually quantified for each organoid.

### 5.20 RNA Isolation and Quantitative Reverse Transcription PCR (RT‐qPCR)

RNA isolation was performed using TRIzol according to the manufacturer's instructions (Thermo Fisher Scientific) with modifications below. Samples were collected in 350 µL TRIzol via scraping for 2D and pooling of whole organoids for 3D cultures. To improve visualization of the RNA pellet, glycogen blue was added prior to RNA precipitation from the aqueous phase with 175 µL isopropanol, which was incubated for 30 min −80°C. Upon subsequent centrifugation at 12 000 × *g* at 4°C for 10 min RNA formed a blue gel‐like pellet. The supernatant was removed, and RNA was washed twice with cold 75% ethanol and air‐dried after a final centrifugation for 10 min at RT. RNA pellets were resuspended in RNAse‐free water and incubated at 58°C for 10 min before measuring the final concentration of RNA using a NanoDrop One.

For RT‐qPCR assays, 1 µg RNA was first reverse‐transcribed to cDNA using Superscript III Reverse Transcriptase First‐Strand Synthesis SuperMix (Thermo Fisher Scientific). The thermal cycler was set to 25°C 10 min, 50°C 30 min, 85°C 5 min, and finally 37°C 20 min incubation with *E. Coli* RNAse H. Subsequent qPCR reactions were performed in 384‐well plates using a ViiA^TM^ 7 384‐plate reader (Thermo Fisher Scientific; thermal profile: 40× (95°C 15 s, 60°C 60 s)). Per well, the reaction contained platinum SYBR green QPCR SuperMix‐UDG (Thermo Fisher Scientific) with 5 µL SYBR green + 1:500 ROX 1:5 diluted cDNA and 2.5 µL each of reverse and forward primer (primer concentrations were 100 nm; an overview of oligonucleotide primer sequences is provided in Annex 1). To validate the stability of reference genes under our experimental conditions, the expression of *GAPDH, RPS9, RPS15A, RPL13A*, and *HPRT* was assessed in HC‐, DMD‐Iso‐, and DMD‐COs at day 15 of cardiac differentiation. No significant differences in *Ct*‐values were observed among groups for any of the tested housekeeping genes (Figure S3B), indicating stable expression across conditions. Based on this validation, −delta *Ct* (−Δ*Ct*) values were calculated by subtracting the *Ct*‐values of the genes of interest from the *Ct*‐values of the *GAPDH* reference gene.

### 5.21 Statistical Analysis

Data are expressed as the mean ± standard deviation (SD). GraphPad Prism 10.4.1 was used to generate all graphs and perform statistical analysis. Depending on the variables of the experiment, two‐tailed unpaired Student's *t‐*test analysis, one‐way ANOVA, and two‐way ANOVA, with significance levels indicated with exact *p*‐values. The absence of these values signifies non‐significant results.

## Funding

V.M. and M.C.S. are supported by The Research Foundation Flanders #1S98725N and FWO #1167225N, respectively). M.S. acknowledges funding from FWO (#G058924N), INTERREG – Euregio Meuse‐Rhine (GYM, Generate Your Muscle 2020‐EMR116), Small Research Infrastructure KU Leuven–BioAssemblyBot 400 (KA/20/088), ERA‐NET ERA4Health CARDINNOV: AmnioSMART #G0GE223N, the Ricerca Finalizzata from the Italian Ministry of Health (RF‐2019‐12369703), and Medium Infrastructure KU Leuven (AKUL/19/34 to GB and MS). H.L.R. thanks KU Leuven Research Council (#C14/21/093) for support. G.B. and H.L.R. are partners of the FWO Scientific Research Network CaSign (W0.019.17N and W0.014.22N). I.P. and A.D. acknowledge the support by Interne Fondsen KU Leuven/Internal Funds KU Leuven grant number C24M/22/058.

## Conflicts of Interest

The authors declare no conflicts of interest.

## Supporting information




**Supporting File 1**: adhm70917‐sup‐0001‐Figurecaptions.docx.

**Supporting File 2**: adhm70917‐sup‐0002‐Figure.zip.

## Data Availability

The data that support the findings of this study are available from the corresponding author upon reasonable request.;
