# Supplementary material for: Patient‐Derived 3D Bioprinted Cardiac Organoid Constructs Reveal Key Pathological Features of Duchenne Muscular Dystrophy
Source: Adv Healthc Mater. 2026 Feb 12;15(16):e04004. doi: 10.1002/adhm.202504004 (PMC13107919; doi:10.1002/adhm.202504004)
Supplement: Supplementary file 1 — Supporting File 1: adhm70917‐sup‐0001‐Figurecaptions.docx. [file ADHM-15-0-s002.docx]

Supporting Information

**Figure S1.**

**A)** Representative immunofluorescence images for α-actinin and cTnT in HC-, DMD-Iso-, and DMD-COs at day 15 of cardiac differentiation**.** Nuclei were counterstained with Hoechst. Scale bar = 50 µm. **B)** Representative immunofluorescence images (top panel) and quantification (bottom panel) for α-SMA, COL1A1, PECAM1, and PLIN1 in HC-, DMD-Iso-, and DMD-COs at day 15 of cardiac differentiation**.** Nuclei were counterstained with Hoechst. Data are representative of three independent experiments (n = 3). Statistical analysis was performed using one-way ANOVA with Tukey’s multiple comparisons. Scale bar = 50 µm. **C)** Selection among the top 10 upregulated KEGG pathways from bulk RNA-seq analysis of DMD-COs compared to DMD-Iso-COs (GSE194297) (n = 3 per condition). **D)** Selected KEGG pathways involved in cardiac remodeling from bulk RNA-seq analysis of DMD-COs compared to DMD-Iso-COs (GSE194297) (n = 3 per condition). **E)** Heatmap of the top 50 genes related to cardiac remodeling, fibrosis, and adipose tissue according to KEGG pathways from bulk RNA-seq analysis of DMD-COs compared to DMD-Iso-COs (GSE194297) (n = 3 per condition).

**Figure S2.**

**A)** Representative brightfield images of DMD #1-, DMD #3-, DMD #5-COs at day 15 of cardiac differentiation. Scale bar = 500 µm. **B)** Representative immunofluorescence images for α-SMA, COL1A1, PECAM1, and PLIN1 in DMD #1-, DMD #3-, DMD #5-COs at day 15 of cardiac differentiation**.** Nuclei were counterstained with Hoechst. Scale bar = 50 µm. **C)** Representative live/dead staining images of DMD #1-, DMD #3-, DMD #5-COs at day 15 of cardiac differentiation. Calcein^+^ cells (green)= living cells; Propidium iodide^+^ cells (red) =dead cells). Scale bar = 250 µm. **D)** Representative flow cytometric analyses (left panel) and quantification (right panel) at day 15 of cardiac differentiation showing the percentage of necrotic (DAPI^+^/AnnexinV^–^) cells in HC-, DMD #1-, DMD #3-, DMD #5-COs. Data are representative of three independent experiments (n = 3, each pooled of ~50 organoids) and expressed as mean ± SD. Statistical analysis was performed using one-way ANOVA with Tukey’s multiple comparisons test. **E)** Average Young’s modulus of HC- and DMDs-COs at day 15 of cardiac differentiation**.** Data are representative of three independent experiments (n = 3) and expressed as mean ± SD. Statistical analysis was performed using Unpaired t test.

**Figure S3.**

**A**) Representative flow cytometric analyses (left panel) and quantification (right panel) at day 15 of cardiac differentiation showing the percentage of ROS-containing (FITC^+^) hiPSC-CMs (SIRPA-PE^+^) in HC-, DMD #1-, DMD #3-, DMD #5-COs. Data are representative of three independent experiments (n = 3, each pooled of ~50 organoids) and expressed as mean ± SD. Statistical analysis was performed using one-way ANOVA with Tukey’s multiple comparisons test. **B**) Gene expression analysis of housekeeping genes (GAPDH, RPS9, RPS15A, RPL13A, HPRT) at days 15 of cardiac differentiation in HC-, DMD-Iso-, and DMD-COs. Data are presented as Ct values. Data are representative of three independent experiments (n = 3, each pooled of ~50 organoids) and expressed as mean ± SD. Statistical analysis was performed using 2way ANOVA with Tukey’s multiple comparisons test. **C**) Gene expression analysis of *NOX4* at day 15 of cardiac differentiation in HC-, DMD #1-, DMD #3-, DMD #5-COs. Each data point is represented as −ΔCt, normalized to the housekeeping gene GAPDH. Data are representative of three independent experiments (n = 3, each pooled of ~50 organoids) and expressed as mean ± SD. Statistical analysis was performed using one-way ANOVA with Tukey’s multiple comparisons test. **D)** Gene expression analysis of *NOX2* and its regulatory subunits (*p22phox*, *p47phox*, *RAC1*, *RAC2*, and *RAC3*) of HC-, DMD-Iso-, DMD-, DMD #1-, DMD #3-, and DMD #5-COs at day 15 of differentiation. Each data point is represented as −ΔCt, normalized to the housekeeping gene GAPDH. Data are representative of three independent experiments (n = 3, each pooled of ~50 organoids) and expressed as mean. Statistical analysis was performed using two-way ANOVA with Dunnett’s multiple comparisons test. *p < 0.05, **p < 0.01, ****p < 0.0001 indicate differences against HC-COs.

**Figure S4.**

**A)** Representative traces of Ca²⁺ transient recordings from Cal520-loaded HC-, DMD-Iso-, and DMD-COs at day 15 of cardiac differentiation, plotted as F/F0(F_basal_) over time. Measurements were performed under electrical stimulation at 0.5 Hz. **B)** Quantification of the percentage of DMD-, DMD-Iso and HC-COs displaying double peaks under unstimulated and electrical pacing conditions at 0.5 and 1Hz (n = 3). **C-H)** Quantification of calcium transient parameters, including: **C)** frequency, **D)** peak-to-peak (P-P) time, **E)** rising slope, **F)** falling slope, **G)** area under the curve (AUC), and **H)** amplitude, in HC-, DMD-Iso-, and DMD-COs under paced stimulation at 0.5 Hz at day 15 of differentiation. Data are representative of three or more independent experiments (n ≥ 3) and expressed as mean ± SD. Statistical analysis was performed using one-way ANOVA with Tukey’s multiple comparisons test. **I)** Heatmap of genes from bulk RNAseq analysis related to action potential (left panel) and related representation (right panel) of upregulated (green) and downregulated (red) markers in DMD-COs compared to DMD-Iso-COs. (n = 3 per condition). **J)** Heatmap of genes from bulk RNAseq analysis related to calcium handling (left panel) and BioRender schematic representation (right panel) of Ca^2+^ pumps and channels upregulated (green) and downregulated (red) in DMD-COs compared to DMD-Iso-COs; (n = 3 per condition). **K)** Heatmap of genes from bulk RNAseq analysis related to fetal cardiac gene reactivation in DMD-COs compared to DMD-Iso-COs; (n = 3 per condition).

**Figure S5.**

**A)** Representative images of live/dead staining of HC-bCOs in 3% alginate-2% gelatin hydrogel at day 0, 10, and 20 pb. Calcein^+^ cells (green)= living cells; Propidium iodide^+^ cells (red) =dead cells. Scale bar = 250 µm. **B)** Representative brightfield images showing the morphology of healthy, isogenic, and dystrophic COs at day 1, 5, 8, 11, and at 14 pb. Scale bar = 500 µm. **C)** Representative images of live/dead staining of HC-, DMD-Iso, and DMD-COs at days 1, 7, and 14 pb. Calcein^+^ cells (green)= living cells; Propidium iodide^+^ cells (red) =dead cells. Scale bar = 250 µm. **D)** Quantification of cell death as percentage of PI^+^ area/ total organoid area in HC-, DMD-Iso-, and DMD-COs at days 1, 7, and 14 post-bioprinting. Data are representative of three independent experiments (n = 3) and expressed as mean ± SD. Statistical analysis was performed using one-way ANOVA with Tukey’s multiple comparisons test.

**Figure S6.**

**A)** Representative brightfield images showing the morphology of DMD #1-, DMD #3-, DMD #5-bCOs at day14 post-bioprinting. Scale bar = 500 µm. **B)** Representative live/dead staining images of DMD #1-, DMD #3-, DMD #5-bCOs at days 1, 7, and 14 pb. Calcein^+^ cells (green)= living cells; Propidium iodide^+^ cells (red) =dead cells. Scale bar = 250 µm. **C)** Quantification of DMD #1-, DMD #3-, DMD #5-COs and bCOs area at days 1, 7, and 14 post-bioprinting. Data are representative of three independent experiments (n = 3) and expressed as mean ± SD. Statistical analysis was performed using one-way ANOVA with Šídák’s multiple comparisons test. **D)** Gene expression analysis of cardiac markers in DMD #1-, DMD #3-, DMD #5-bCOs at days 1, 7, and 14 post bioprinting and their respective hiPSC lines. Data are representative of four independent experiments (n = 4, each pooled of ~50 organoids).

**Figure S7.**

**A)** Heatmap of genes from bulk RNAseq analysis related to mesenchymal drift in DMD-COs compared to DMD-Iso-COs. (n = 3 per condition).
