# Supplementary figures and images for: Patient‐Derived 3D Bioprinted Cardiac Organoid Constructs Reveal Key Pathological Features of Duchenne Muscular Dystrophy
Source: Adv Healthc Mater. 2026 Feb 12;15(16):e04004. doi: 10.1002/adhm.202504004 (PMC13107919; doi:10.1002/adhm.202504004)

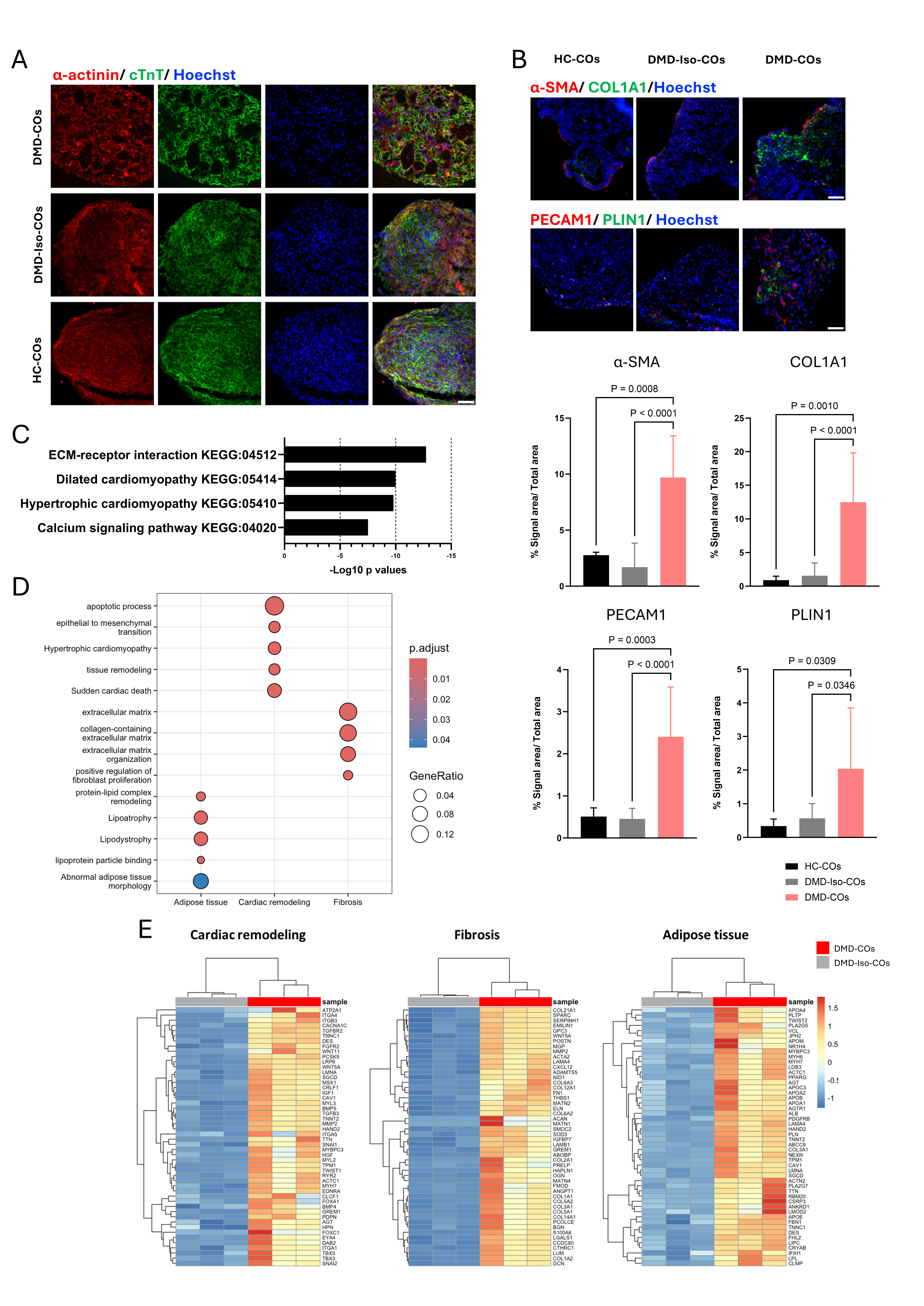

Supplement: Supplementary file 2 — Supporting File 2: adhm70917‐sup‐0002‐Figure.zip. [file ADHM-15-0-s001.zip › adhm70917-sup-0002-Figure/adhm70917-sup-0001-FigureS1.png]

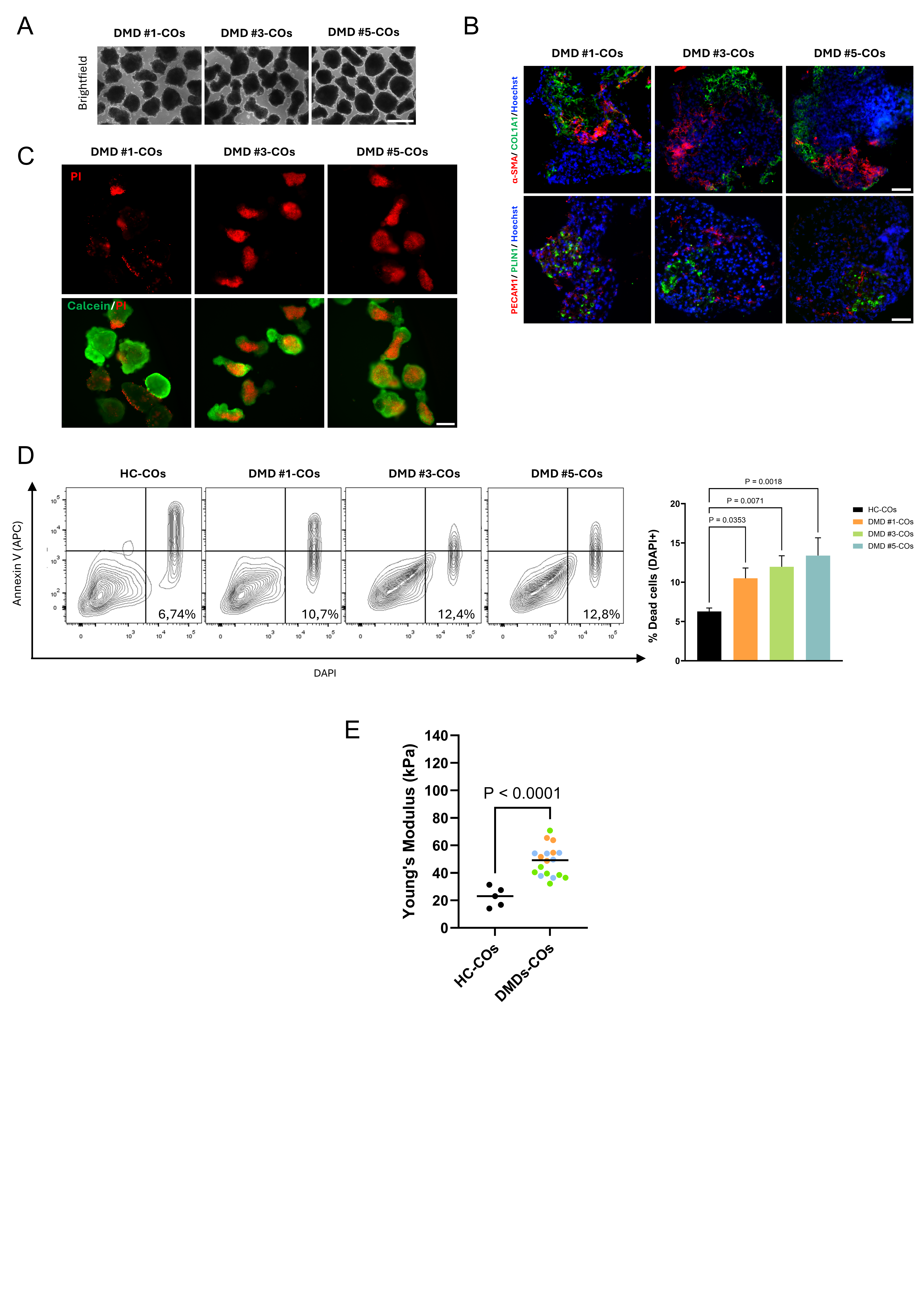

Supplement: Supplementary file 2 — Supporting File 2: adhm70917‐sup‐0002‐Figure.zip. [file ADHM-15-0-s001.zip › adhm70917-sup-0002-Figure/adhm70917-sup-0002-FigureS2.png]

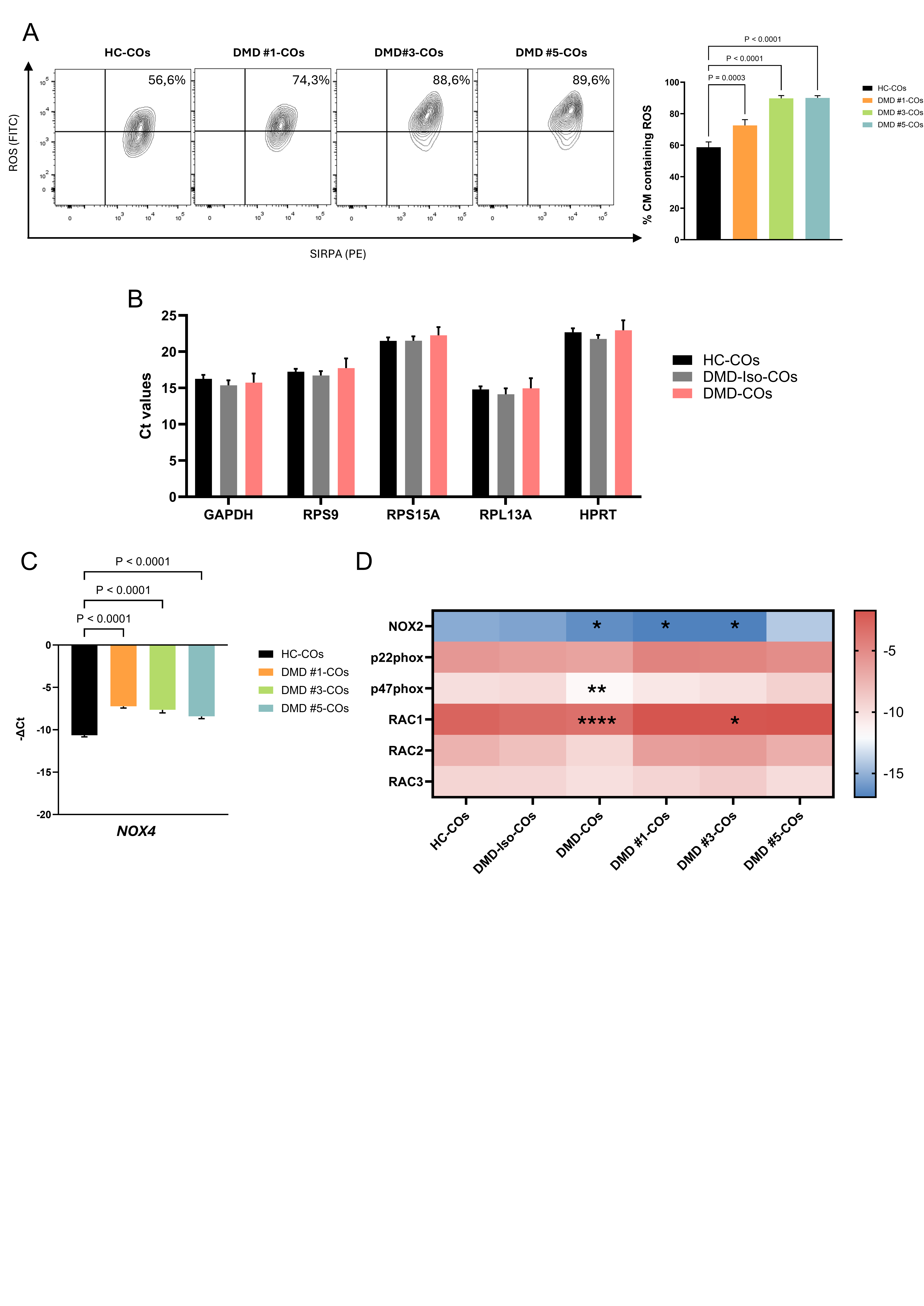

Supplement: Supplementary file 2 — Supporting File 2: adhm70917‐sup‐0002‐Figure.zip. [file ADHM-15-0-s001.zip › adhm70917-sup-0002-Figure/adhm70917-sup-0003-FigureS3.png]

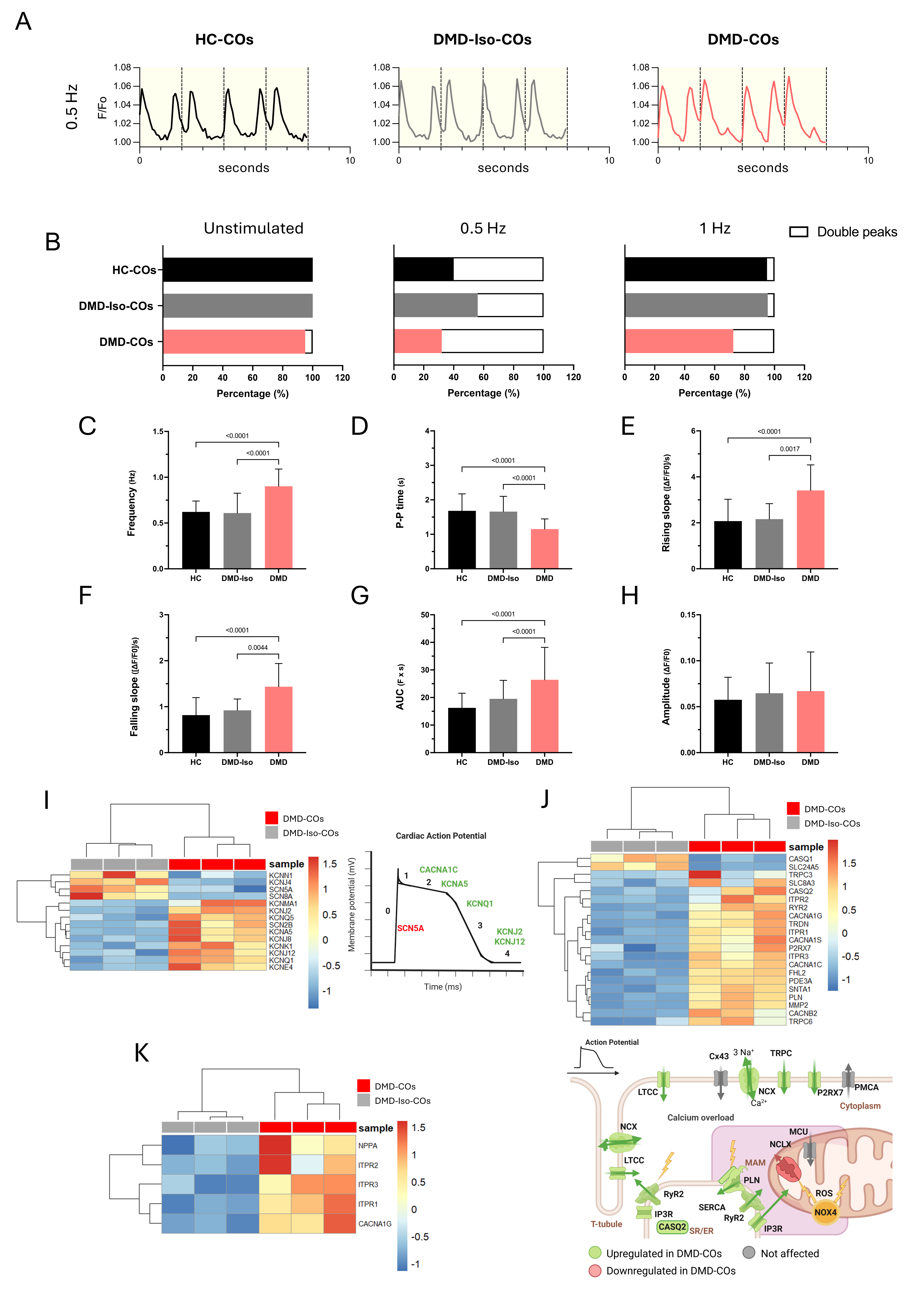

Supplement: Supplementary file 2 — Supporting File 2: adhm70917‐sup‐0002‐Figure.zip. [file ADHM-15-0-s001.zip › adhm70917-sup-0002-Figure/adhm70917-sup-0004-FigureS4.png]

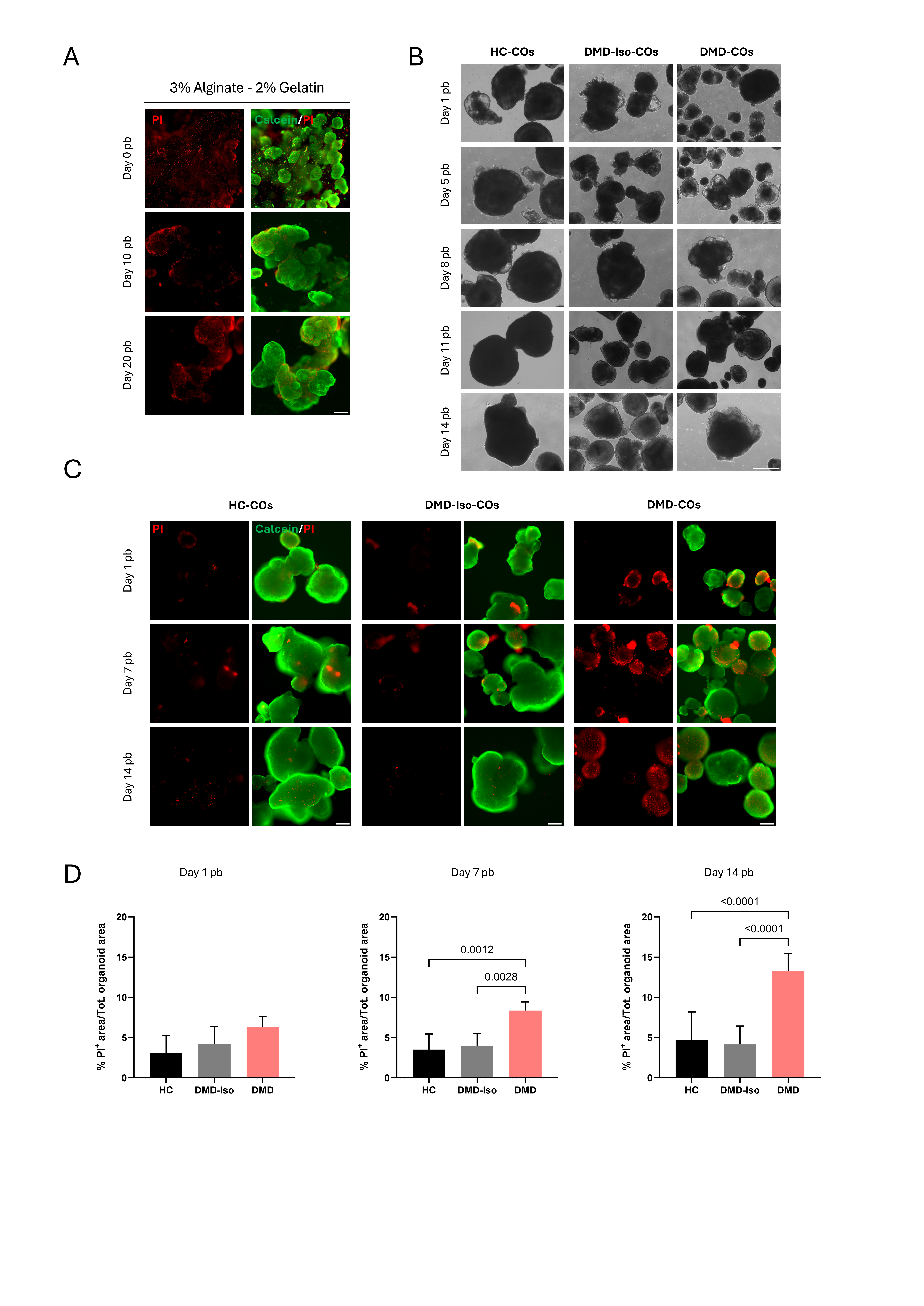

Supplement: Supplementary file 2 — Supporting File 2: adhm70917‐sup‐0002‐Figure.zip. [file ADHM-15-0-s001.zip › adhm70917-sup-0002-Figure/adhm70917-sup-0005-FigureS5.png]

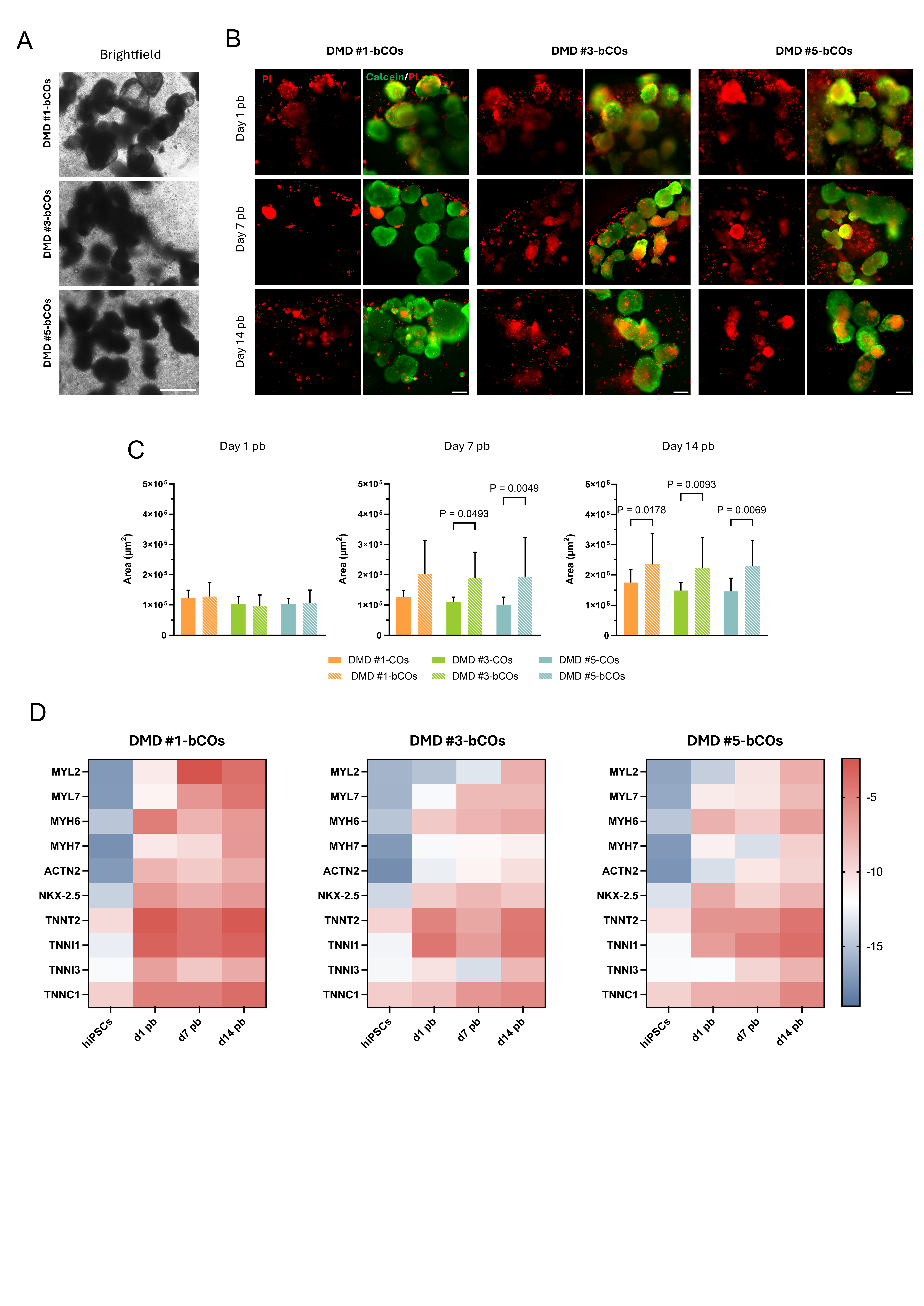

Supplement: Supplementary file 2 — Supporting File 2: adhm70917‐sup‐0002‐Figure.zip. [file ADHM-15-0-s001.zip › adhm70917-sup-0002-Figure/adhm70917-sup-0006-FigureS6.png]

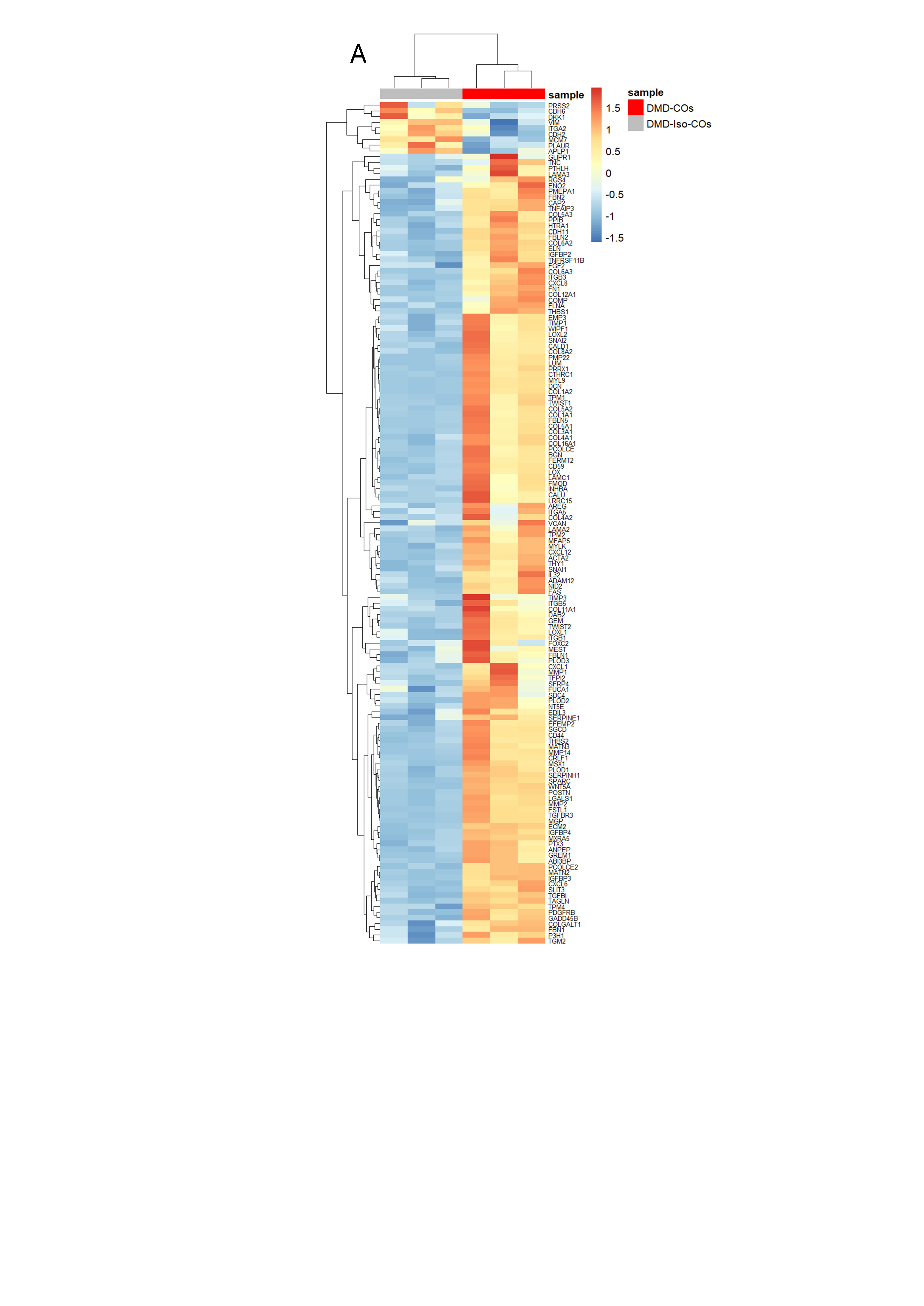

Supplement: Supplementary file 2 — Supporting File 2: adhm70917‐sup‐0002‐Figure.zip. [file ADHM-15-0-s001.zip › adhm70917-sup-0002-Figure/adhm70917-sup-0007-FigureS7.png]
